# The Cuproptosis-Related Long Noncoding RNA Signature Predicts Prognosis and Tumour Immune Analysis in Osteosarcoma

**DOI:** 10.1155/2022/6314182

**Published:** 2022-11-02

**Authors:** Jinxia Jiang, Dejun Chu, Xingming Lai, Li Liu, Jun Tao

**Affiliations:** ^1^Department of Orthopedics, The Second Affiliated Hospital of Nanchang University, China; ^2^Department of Sports Medicine, Xiangya Hospital, Central South University, China; ^3^Department of Orthopedics, Guilin People's Hospital, China; ^4^Department of Gastrointestinal Surgery, The First Affiliated Hospital of Nanchang University, China

## Abstract

**Background:**

Cuprotopsis is a type of programmed cell death discovered in recent years. Long noncoding RNAs (lncRNAs) play an important regulatory role in programmed cell death. The effect of cuproptosis-related lncRNAs on osteosarcoma is unknown. Our work, based on cuproptosis-related lncRNAs, proposes a gene signature to assess the prognosis of patients with osteosarcoma.

**Methods:**

Osteosarcoma gene expression data from The Cancer Genome Atlas (TCGA), clinical features of osteosarcoma and RNA sequencing data of normal adipose tissue were obtained from the UCSC Xena database. A cuproptosis-related lncRNA risk model was established to calculate the risk score. At the same time, cluster analysis, clinicopathological analysis, functional enrichment analysis, and prediction of compounds with potential therapeutic value were evaluated. We analyzed whether there was a correlation between the risk score and tumour immunity. RT-qPCR was used to verify the expression level of lncRNA.

**Results:**

Nine lncRNAs (AC124798.1, AC006033.2, AL450344.2, AL512625.2, LINC01060, LINC00837, AC004943.2, AC064836.3, and AC100821.2) were identified to create a risk model and indicate the prognosis of patients with osteosarcoma. The high-risk group had a worse prognosis than the low-risk group. Analysis of clinicopathological features, principal component analysis, receiver operating characteristic curve, c-index curve, and comparative analysis of models proved that the model is reliable. Functional enrichment analysis suggests that the risk score may correlate with cell energy metabolism and tumour-related biological function. Three potentially therapeutic compounds have been predicted. These analyses may be beneficial to the treatment of osteosarcoma in the future. RT-qPCR verified the expression level of three lncRNA (LINC01060, NKILA, and SNHG8).

**Conclusions:**

Cuproptosis-related lncRNAs have a strong relationship with osteosarcoma patients. Nine lncRNA models can effectively forecast the prognosis of osteosarcoma and may play a significant role in the individualized treatment of osteosarcoma patients in the future.

## 1. Introduction

Osteosarcoma originates from primitive mesenchymal cells [1] and usually occurs in the epiphysis of the femur, humerus, and tibia. It is the most common malignant bone tumour in adolescents [2]. The malignancy of osteosarcoma is high; the survival rate of osteosarcoma with metastasis is only 20%, and there has been little progress in effective treatment in recent years [3]. Conventional treatment is both inaccurate and may bring serious side effects [4–6]. Therefore, the use of effective predictive models to accurately stratify osteosarcoma patients is helpful for their treatment. In recent years, open gene expression datasets have provided an opportunity to develop new prediction tools based on prediction-related genes.

Copper is an important factor involved in cell metabolism, and maintaining a certain concentration of copper is conducive to cell growth [7]. However, too much copper can lead to copper poisoning and cell death. New research has shown that copper-dependent death occurs through direct binding of copper to lipidated components of the tricarboxylic acid (TCA) cycle, triggering an unusual mode of cell death [8]. The study used a synthetic molecule that binds to copper present in the environment and brings it into cells to induce cell death. Kahlson and Dixon suggest that this different mechanism of cell death is called cuproptosis [9]. This mechanism may explain the pathological mechanism of hereditary copper overload and how copper toxicity can be used to treat cancer. For tumour cells that undergo nearly no apoptosis, this newly discovered mode of cell death may be helpful for the treatment of tumours.

Long noncoding RNA has long been considered an important factor in tumour regulation [10], and some references have indicated that lncRNAs may affect the regulation of cuproptosis [11, 12]. At present, there is no published research on cuproptosis-related lncRNAs in osteosarcoma. In our study, we screened cuproptosis-related lncRNAs found in osteosarcoma based on The Cancer Genome Atlas (TCGA) database [13] and the UCSC Xena website [14] and established a prognostic cuproptosis-related lncRNA model. The purpose of this research is to take advantage of bioinformatics to study the relationship between cuproptosis-related lncRNAs and osteosarcoma and to construct a comprehensive prediction model for individual osteosarcoma prognosis, which may have therapeutic significance in the future.

## 2. Methods

### 2.1. Data Collection

The cuproptosis-related genes were screened by consulting published articles [8, 9]. “TCGA-TARGET-OS”, the RNA sequencing data of osteosarcoma were obtained from The Cancer Genome Atlas (TCGA) website (https://portal.gdc.cancer.gov/repository); clinical features of osteosarcoma and RNA sequencing data of normal adipose tissue were obtained from the UCSC xena website (http://xena.ucsc.edu/). All data have been downloaded on May 10, 2022. Including 83 RNA sequencing data of osteosarcoma, 80 matching clinical features of osteosarcoma, and 80 RNA sequencing data of random normal adipose tissue. The data were standardized by fragment per kilobase million (FPKM) [15]. The KEGG dataset was acquired from the GSEA website (http://www.gsea-msigdb.org/gsea/) [16]. All data in this study come from the public database, so this study does not need to pass the ethical review.

### 2.2. Selection of Cuproptosis-Related lncRNAs

Cuproptosis-related lncRNAs were screened by Pearson test. The screening criteria are |Pearson R| > 0.4 and *P* < 0.001. The “limma” package [17] was used for computation, “ggplot2”, “ggalluvial”, and “dplyr” packages were used for visualization and draw Sankey diagrams. Use volcano plot to reflect differential expression of cuproptosis-related lncRNAs in osteosarcoma, when |logFC| > 1 and *P* < 0.05, it was considered that there is a difference in expression level.

### 2.3. GO and KEGG Analysis

The biological functions of cuproptosis related lncRNAs were investigated using Gene Ontology (GO) and Kyoto Encyclopedia of Genes and Genomes (KEGG). The GO analysis consists of three parts: Biological Process (BP), Cellular Component (CC), and Molecular Function (MF); it reflects the possible molecular function of the gene product, the cellular environment, and the biological process involved. “clusterProfiler”, “http://org.Hs.eg.db”, “enrichplot”, and “ggplot2” packages were used for these analyses and draw bubble chart.

### 2.4. Prognostic Cuproptosis-Related lncRNAs and Cluster Analysis

Univariate cox analysis [18] was utilized to pick out prognostic cuproptosis-related lncRNAs; the “survival” package was used, and *P* < 0.05 was set as a significant filtering condition; forest plots were drawn to visualize the outcomes.

In order to find the differential typing of osteosarcoma, we tried to type cuproptosis-related lncRNAs by use cluster analysis. “limma” and “ConsensusClusterPlus” packages [19] were used for cluster analysis, “survminer”, “survival”, and “pheat map” packages to show the results and draw the survival curve and the heat map of clinicopathological features (age, gender, and metastatic). Samples lacking relevant clinical data had been deleted before analysis.

### 2.5. Constructing the Risk Model

The osteosarcoma samples with clinical data were equally grouped into training group and testing group. The least absolute shrinkage and selection operator (LASSO) regression [20] was used to build a model, then calculate the risk score for each sample [21]. The risk scoring rule was: risk score = *Σ* (Exp[lncRNA] × coef[lncRNA]). Coef[lncRNA] represented the review coefficient, and Exp[lncRNA] reflected the expression level of corresponding lncRNA. Each sample was classified as high-risk or low-risk based on the median risk score.

Heatmap of the correlation between risk lncRNAs and cuproptosis-related genes, Kaplan-Meier curve [22] for overall survival (OS), risk curve, and heat map of risk lncRNAs between high- and low-risk were drawn. We showed these indicators in three levels: all samples, training group samples, and testing group samples. “survival”, “caret”, “glmnet”, “survminer”, “timeROC”, “tidyverse”, “ggplot2”, “ggExtra”, and “pheatmap” packages were utilized for the above analysis and visualized the results. (^∗^ if *P* < 0.05, ^∗∗^ if *P* < 0.01, and ^∗∗∗^ if *P* < 0.001).

### 2.6. Independent Prognostic Analysis and Clinicopathological Features

Univariate and multivariate cox analyses were performed on all samples to screen for factors (age, metastasis, gender, and risk score) that might be independent prognostic factors for osteosarcoma. The “survival” package was used for independent prognostic analysis. Different clinicopathological features (gender, age, and metastatic) were selected separately to explore whether there was a distinction in survival between high- and low-risk samples. A bar chart was drawn to explore the difference of metastatic relative content between high- and low-risk samples. “plyr”, “ggplot2”, “survival”, “ggpubr”, and “survminer” packages were applied to analysis.

### 2.7. Validation of the Risk Model

To verify the accuracy of the risk model, Principal component analysis (PCA), ROC curves [23], ROC curve of multiple indicators, and concordance index (C-index) curves were drawn. The “dplyr”, “rms”, “survival”, “pec”, “survminer”, “limma”, and “timeROC” packages were used for analysis to help validate the validity of the risk model.

From the published articles, we selected two validated literatures that used the same osteosarcoma sample data to build risk models. We can verify the validity of our model by comparing the AUC values. It was worth noting that the AUC values compared here were based on the comparison of the entire osteosarcoma samples. “limma”, “survival”, “survminer”, and “timeROC” packages were used for model comparison.

### 2.8. Nomogram Prediction Model

Nomogram [24] was mapped to predict survival in patients with osteosarcoma, and used a five-year calibration curve to proof the effectiveness of the nomogram. “survival”, “regplot”, and “rms” packages were used for the analysis. The nomogram was constructed by using gender, age, metastatic, and risk score as factors. (^∗^ if *P* < 0.05, ^∗∗^ if *P* < 0.01, and ^∗∗∗^ if *P* < 0.001).

### 2.9. Prediction of Potentially Therapeutic Compounds

Some potential therapeutic compounds have been predicted, which may be beneficial to the treatment of osteosarcoma over the next few years. The IC50 values of compounds retrieved from the GDSC website (https://www.cancerrxgene.org/) were calculated to forecast the sensitivity of osteosarcoma patients to these compounds. “limma”, “ggpubr”, “pRRophetic”, and “ggplot2” packages were used for analysis.

### 2.10. Gene Set Enrichment Analysis and Gene Set Variation Analysis

We used downloaded KEGG gene set data to conduct gene set enrichment analysis (GSEA) enrichment analysis in two risk groups. Gene set variation analysis (GSVA) enrichment analysis was performed to understand the relationship between the expression of risk lncRNAs, risk score, and biological function. These analyses are helpful for us to understand the relationship between risk score and biological function. “limma”, “http://org.Hs.eg.db”, “clusterProfiler”, “enrichplot”, “GSEABase”, “GSVA”, “reshape2”, and “ggplot2” packages were utilized for GSEA and GSVA enrichment analysis. (^∗^ if *P* < 0.05, ^∗∗^ if *P* < 0.01, and ^∗∗∗^ if *P* < 0.001).

### 2.11. Tumour Immune Analysis

Tumour immunity is the focus of tumour research at present. We scored the immunity of the two groups (including ESTIMATE score, Immune score, and Stromal score) to assess the difference of immune infiltration degree about the two risk groups.

The differential expression of 47 immune checkpoint genes (IDO1, LAG3, CTLA4, TNFRSF9, ICOS, CD80, PDCD1LG2, TIGIT, CD70, TNFSF9, ICOSLG, KIR3DL1, CD86, PDCD1, LAIR1, TNFRSF8, TNFSF15, TNFRSF14, IDO2, CD276, CD40, TNFRSF4, TNFSF14, HHLA2, CD244, CD274, HAVCR2, CD27, BTLA, LGALS9, TMIGD2, CD28, CD48, TNFRSF25, CD40LG, ADORA2A, VTCN1, CD160, CD44, TNFSF18, TNFRSF18, BTNL2, C10orf54, CD200R1, TNFSF4, CD200, and NRP1) between high- and low-risk groups were also analyzed. “reshape2”, “limma”, “ggpubr”, and “ggplot2” packages were utilized for this analysis.

We used ssGSEA algorithm to analyze the differences in the enrichment of different immune functions in high and low risk populations. “GSVA”, “limma”, “GSEABase”, “reshape2”, and “pheatmap” packages were used for immune function analysis and corresponding heatmaps were drawn. (^∗^ if *P* < 0.05, ^∗∗^ if *P* < 0.01, and ^∗∗∗^ if *P* < 0.001).

### 2.12. Quantitative Real-Time Polymerase Chain Reaction

Osteoblast line hFOB, osteosarcoma cell line Saos-2, and U2OS were supplied by the Chinese Academy of Sciences' Shanghai Cell Bank. Total RNA was extracted using TRIzol (Invitrogen, Carlsbad, CA, USA) reagent according to the manufacturer's instructions. lncRNA reverse transcription was performed using PrimeScript RT Master Mix (Takara, Japan). RT-qPCR was performed using SYBR Green (Takara, Japan) according to the manufacturer's instructions. Results were normalized to GADPH expression and calculated according to the 2^−ΔΔCT^ method (mean ± SD) with three replications. Considering that the expression of many lncRNAs was very low, we chose three lncRNAs (LINC01060, SNHG8, and NKILA) that were relatively easy to obtain results for qRT–PCR verification. The primer sequences are shown as follows: LINC01060 (Forward: TCAAGCGCATCTTCCACACT, Reverse: AGGATGGCATCAGTGGCAAA), SNHG8 (Forward: ACATCAAGCCCAAATCTGCTC, Reverse: TTCCTGGTCCCAGTCTTGGC), NKILA (Forward: CTTTGGAGGAGTCCAAGCGT, Reverse: GTGGCTCCAAGAGTGAGCTT), and GAPDH (Forward: CCCACTCCTCCACCTTTGAC, Reverse: CCACCACCCTGTTGCTGTAG). (^∗^ if *P* < 0.05, ^∗∗^ if *P* < 0.01, and ^∗∗∗^ if *P* < 0.001).

### 2.13. Statistical Analysis

R software (version 4.1.2) was used for statistical analysis and result visualization. The differential expressions were authenticated using the Benjamini–Hochberg technique. The mRNA level of pyroptosis-related lncRNAs was determined using the Mann–Whitney *U* test. The Student *t*-test was used to determine the distinction between the two groups. The chi-square test was applied to compare the categorization variables in the training and testing tests. The Pearson correlation test was applied to analyze the relationship between subtypes, clinicopathological variables, risk score, immunological check inhibitors, and immune infiltration levels. For survival analysis, the Kaplan–Meier technique was used, along with a two-sided log-rank test.

## 3. Result

### 3.1. Cuproptosis-Related lncRNAs and Biological Function Analysis

**Figure 1:**
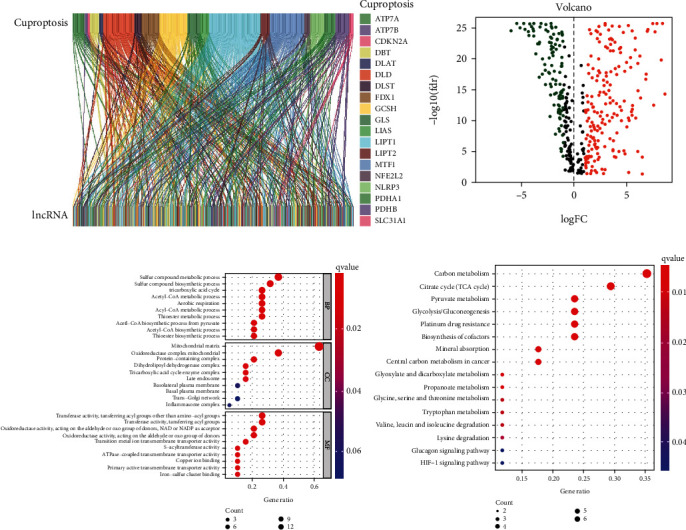
Cuproptosis-related lncRNAs and function analysis. (a) Sankey diagram of cuproptosis-related genes and lncRNAs, (b) Volcano plot of cuproptosis-related lncRNAs, (c) GO analysis of cuproptosis-related lncRNAs (including three functional parts: BP, CC, and MF), (d) KEGG analysis of cuproptosis-related lncRNAs.

Nineteen cuproptosis-related genes (NFE2L2, NLRP3, ATP7B, ATP7A, SLC31A1, FDX1, LIAS, LIPT1, LIPT2, DLD, DLAT, PDHA1, PDHB, MTF1, GLS, CDKN2A, DBT, GCSH, and DLST) were selected from the published literature and included in our study. 431 cuproptosis-related lncRNAs were identified by Pearson analysis (∣Pearson *R* | >0.4 and *P* < 0.001) **(**Figure 1(a)). 109 lncRNAs were downregulated in osteosarcoma samples, and 185 lncRNAs were upregulated in osteosarcoma samples (∣logFC |   > 1 and *P* < 0.05) (Figure 1(b)). GO analysis showed that cuproptosis-related lncRNAs had a strong relationship with cell metabolism in BP, had a strong relationship with cellular energy metabolism in CC, and had a strong relationship with cell redox and energy metabolism in MF (Figure 1(c)). KEGG analysis showed that cuproptosis-related lncRNAs was correlated with cell metabolism, energy, drug resistance, and other biological functions (Figure 1(d)).

**Figure 2:**
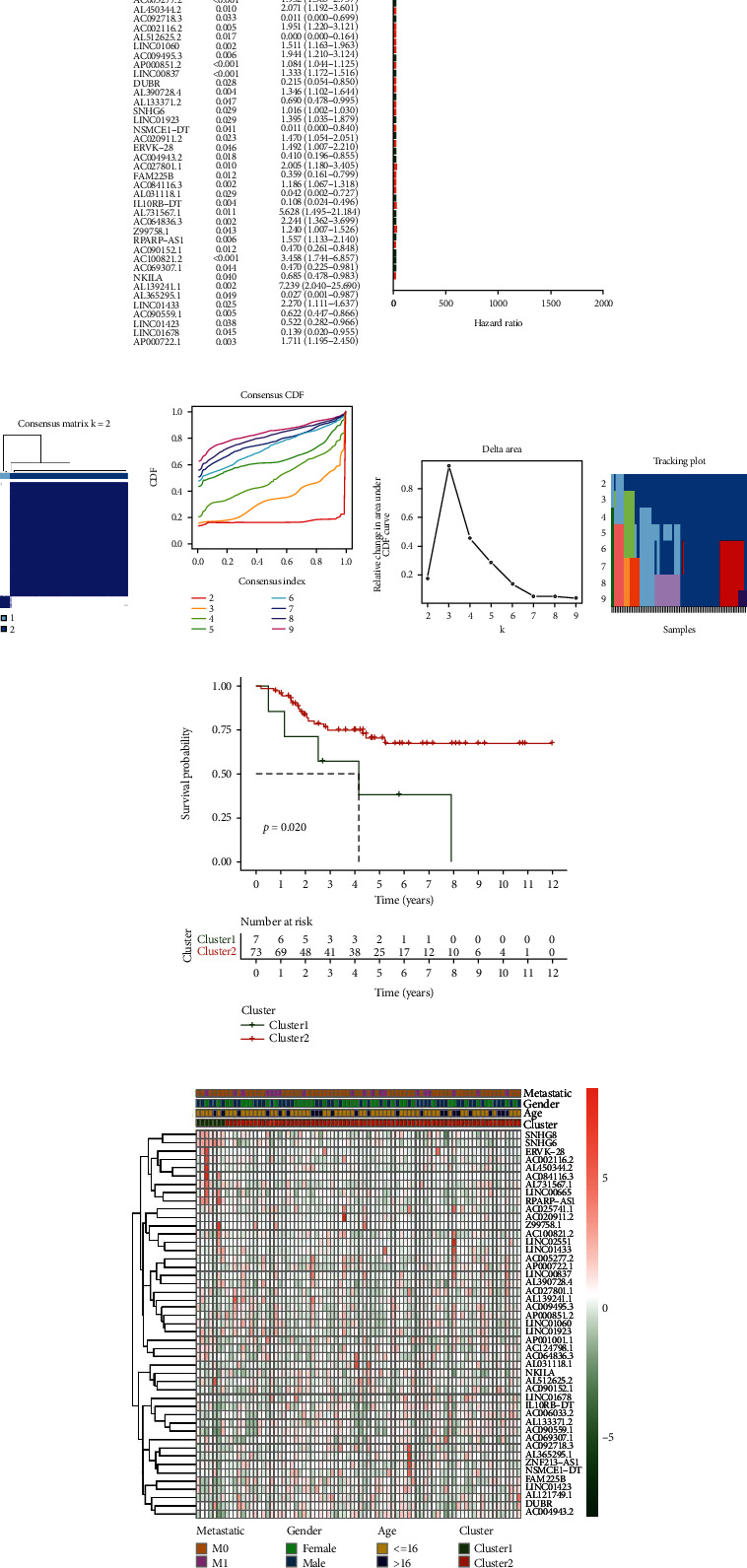
Prognostic cuproptosis-related lncRNAs and cluster analysis. (a) Forest plot based on univariate cox hazard analysis of cuproptosis-related lncRNAs, (b) Consensus clustering matrix for *k* = 2, (c) Consensus clustering distribution function (CDF) for *k* = 2 to 9, (d) Area under CDF curve increment for *k* = 2 to 9, (e) Tracking plot for *k* = 2 to 9, (f) Kaplan-Meier curves of OS in two clusters, (g) Heatmap of cuproptosis-related lncRNAs expression and clinicopathologic features in two clusters.

Univariate cox analysis (*P* < 0.05) was utilized to select 47 prognostic cuproptosis-related lncRNAs: LINC02551, AP001001.1, AL121749.1, LINC00665, AC025741.1, AC124798.1, ZNF213-AS1, AC006033.2, SNHG8, AC005277.2, AL450344.2, AC092718.3, AC002116.2, AL512625.2, LINC01060, AC009495.3, AP000851.2, LINC00837, DUBR, AL390728.4, AL133371.2, SNHG6, LINC01923, NSMCE1-DT, AC020911.2, ERVK-28, AC004943.2, AC027801.1, FAM225B, AC084116.3, AL031118.1, IL10RB-DT, AL731567.1, AC064836.3, Z99758.1, RPARP-AS1, AC090152.1, AC100821.2, AC069307.1, NKILA, AL139241.1, AL365295.1, LINC01433, AC090559.1, LINC01423, LINC01678, and AP000722.1. The forest plot was used to describe this result (Figure 2(a)).

### 3.2. Consensus Cluster Analysis of Prognostic Cuproptosis-Related lncRNAs

We conducted cluster analysis based on osteosarcoma samples with clinical information through 47 prognosis-related lncRNAs. When the control cluster variable K changes from 2 to 9, we found that the intragroup correlation was highest and the intergroup correlation lowest when *k* = 2 (Figures 2(b-e)). Survival analysis revealed that there were significant survival differences between the two subgroups (Figure 2(f)), but there was no significant correlation between the expression of prognostic lncRNA and clinical features (Figure 2(g)). Among the two clusters, cluster 1 has only 7 samples; the scarcity of the number of samples greatly reduces the credibility of the results. More samples will help to improve the credibility of cluster analysis, which is also the research direction that we need to work hard in the future.

### 3.3. Construction of Risk lncRNAs Model

**Figure 3:**
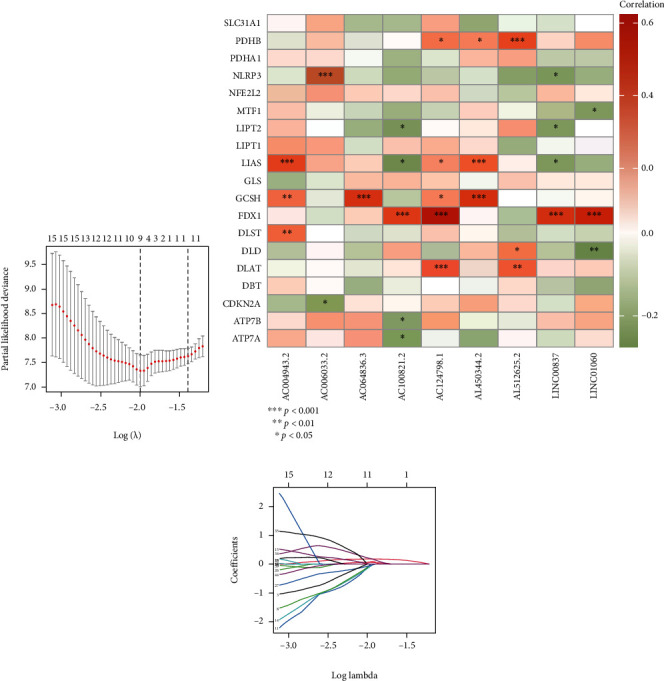
Construction of prognostic model. (a) LASSO coefficient profiles of the cuproptosis-related lncRNAs, (b) Heatmap of the correlation between risk lncRNAs and cuproptosis-related genes, (c) Partial likelihood deviance of different numbers of variables revealed by the LASSO regression model (^∗^ if *P* < 0.05, ^∗∗^ if *P* < 0.01, and ^∗∗∗^ if *P* < 0.001).

Eighty osteosarcoma samples with clinical information were randomly separated into two groups according to the ratio of 1 : 1. Based on 47 prognostic cuproptosis-related lncRNAs, a risk model with 9 lncRNAs (AC124798.1, AC006033.2, AL450344.2, AL512625.2, LINC01060, LINC00837, AC004943.2, AC064836.3, and AC100821.2) was constructed by LASSO regression (Figures 3(a) and 3(c)). Risk score for each osteosarcoma sample, risk score = (0.065762645∗AC124798.1 exp.) + (−0.109837226∗AC006033.2 exp.) + (−0.115274786∗AL450344.2 exp.) + (−0.1512732∗AL512625.2 exp.) + (0.021443894∗LINC01060 exp.) + (0.179123438∗LINC008374 exp.) + (−0.08468658∗AC004943.2 exp.) + (0.212243158∗AC064836.3 exp.) + (0.054191142∗AC100821.2 exp.). According to the median risk score, each sample was classified as high-risk or low-risk. A heatmap reflecting the correlation between risk lncRNAs and cuproptosis-related genes was also drawn (Figure 3(b)).

**Figure 4:**
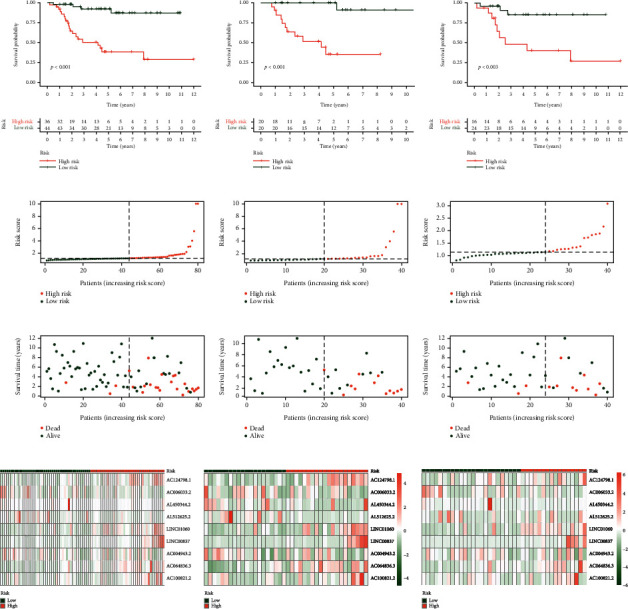
Survival curve and risk curve. (a) Kaplan-Meier curve for OS in all tumour samples, (b) Kaplan-Meier curve for OS in the training group, (c) Kaplan-Meier curve for OS in the testing group, (d) Risk score distribution in all tumour samples, (e) Risk score distribution in the training group, (f) Risk score distribution in the testing group, (g) OS statu in all tumour samples, (h) OS statu in the training group, (i) OS statu in the testing group, (j) Heatmap of risk lncRNAs expression in all tumour samples, (k) Heatmap of risk lncRNAs expression in the training group, (l) Heatmap of risk lncRNAs expression in the training group.

Survival analysis showed that patients in the low-risk group had a better prognosis than those in the high-risk group (Figures 4(a–c)). The risk curve confirms that the prognosis of patients with osteosarcoma improves as the risk score decreases (Figures 4(d–i)). The expression heatmap of risk lncRNAs between high- and low-risk groups were also drawn (Figures 4(j–l)). These analyses indicate that the risk score of the model sample was inversely proportional to the prognosis of patients with osteosarcoma. This conclusion may be beneficial to the treatment of osteosarcoma patients in the days to come.

### 3.4. Independent Prognostic Analysis and Clinicopathological Features

**Figure 5:**
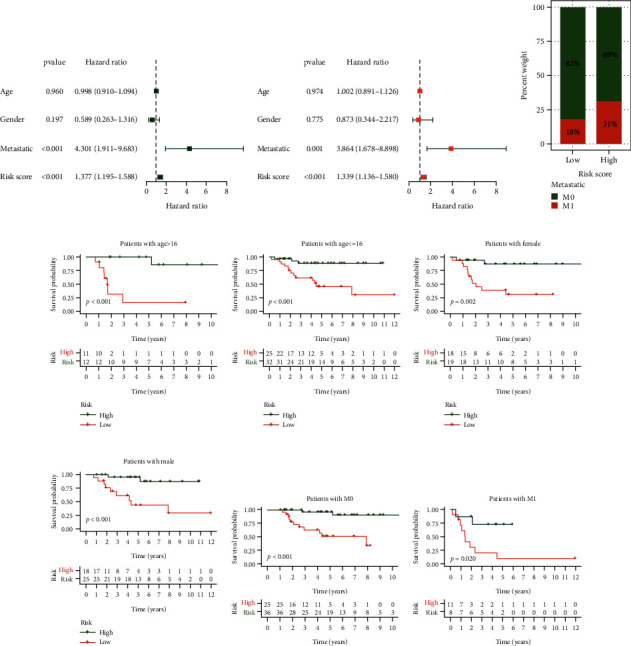
Independent prognostic analysis and clinicopathological features. (a) Univariate independent prognostic analysis in all tumour samples, (b) Multivariate independent prognostic analysis in all tumour samples, (c) Bar chart of the proportion of different metastatic in two risk groups, (d) Kaplan-Meier curve of patients with age >16, (e) Kaplan-Meier curve of patients with age < = 16, (f) Kaplan-Meier curve of patients with female, (g) Kaplan-Meier curve of patients with male, (h) Kaplan-Meier curve of patients with M0, (i) Kaplan-Meier curve of patients with M1.

Univariate and multivariate cox analysis was applied to analyze independent prognostic factors. Metastatic and risk score can be known as independent prognostic factors to analyze the prognosis of patients with osteosarcoma (Figures 5(a) and 5(b)). Between the two risk groups, the relative number of metastatic patients in the low-risk group was significantly lower than that in the high-risk group (Figure 5(c)). This suggests that the risk score has a good effect on forecasting the prognosis of patients with osteosarcoma. Survival analysis of different clinicopathological features (age, gender, and metastatic) also showed that low-risk patients had a better prognosis than high-risk patients (Figures 5(d–i)). This result also implies that the risk model of this study is accurate.

### 3.5. Principal Component Analysis

**Figure 6:**
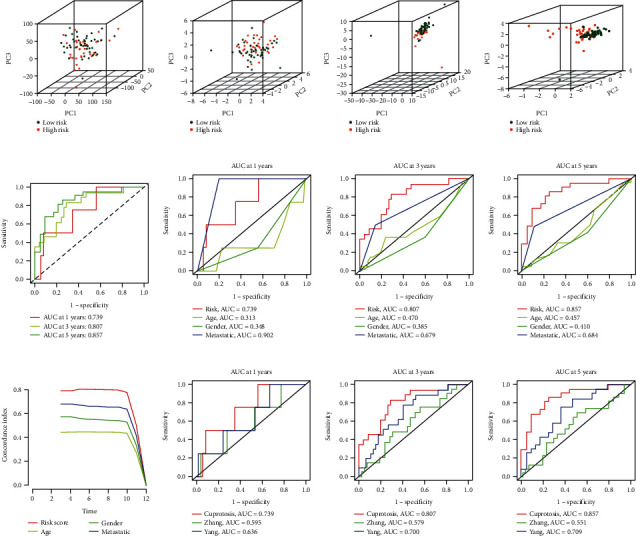
Verification of risk model validity. (a) PCA of all genes, (b) PCA of cuproptosis-related genes, (c) PCA of cuproptosis-related lncRNAs, (d) PCA of risk lncRNAs, (e) ROC curve in all tumour samples, (f) Multi-index ROC curve at 1 years, (g) Multi-index ROC curve at 3 years, (h) Multi-index ROC curve at 5 years, (i) C-index curve in all tumour samples, (j) The ROC curve compared with Zhang and Yang's model at 1 years, (k) The ROC curve compared with Zhang and Yang's model at 3 years, (l) The ROC curve compared with Zhang and Yang's model at 5 years.

PCA was used to assess the differences in four expression profiles (all genes, cuproptosis-related genes, cuproptosis-related lncRNAs, and risk lncRNAs) between two groups. The result was obvious that the risk lncRNAs have better separation ability in the diagram (Figures 6(a–d)). This confirms the good ability of our risk model to distinguish the risk of patients with osteosarcoma and verifies the reliability of model.

### 3.6. Model ROC Curve and C-Index Curve

ROC curve and c-index curve were considered to represent the validity of the risk model. In our research, the 1-year, 3-year and 5-year ROC curves and multi-index ROC curves (risk, age, gender, and metastatic) of all samples were drawn. The AUCs of all samples were 0.739 at 1-years, 0.807 at 3-years, and 0.857 at 5-years (Figure 6(e)). Compared with age and gender, the risk model has obvious prediction advantages. Compared with the AUC value of metastatic, although the risk model was lower than metastatic in 1-year prediction, but it was better than metastatic in 3-year and 5-year prediction (Figures 6(f–h)). The results of c-index curve also confirmed the effectiveness of the risk model (Figure 6(i)).

### 3.7. Model Comparison

The study of Zhang et al. [25] and Yang et al. [26] were included into our study to compare the AUC values between our model and their models. By comparing the AUC values of 1-year, 3-years, and 5-years between models, we found that the model in our study was more effective (Figures 6(j–l)). This showed that our model has higher reliability than the validated model (Zhang and Yang).

### 3.8. Nomogram Prediction

**Figure 7:**
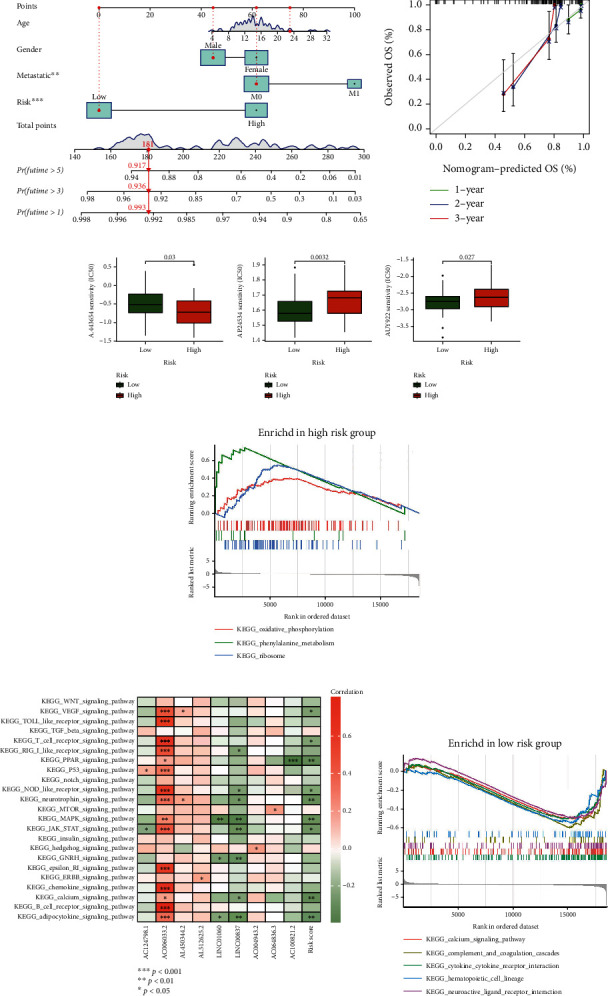
Nomogram prediction, prediction of potentially therapeutic compounds, GSEA and GSVA analysis. (a) The nomogram predicts the probability of the 1-, 3-, and 5-year OS, (b) The calibration plot of the nomogram predicts the probability of the 1-, 3-, and 5-year OS, (c–e) Prediction of potentially therapeutic compounds, (c) A.443654, (d) AP.24534, (e) AUY922, (f) GSEA enrichment analysis of high-risk group, (g) GSVA enrichment analysis of risk lncRNAs, (h) GSEA enrichment analysis of low-risk group (^∗^ if *P* < 0.05, ^∗∗^ if *P* < 0.01, and ^∗∗∗^ if *P* < 0.001).

A nomogram diagram was drawn to predict the prognosis of individual osteosarcoma patients (Figure 7(a)). The calibration plot of the nomogram proved its effectiveness (Figure 7(b)). This analysis may be helpful to the individual diagnosis and treatment of osteosarcoma in the future.

### 3.9. Prediction of Potential Therapeutic Compounds

Using “pRRophetic” package to screen potential compounds with therapeutic value for osteosarcoma by comparing the IC50 values of different compounds between the two risk groups. Three compounds (A.443654, AP.24534, and AUY922) showed significant differences in drug sensitivity between high- and low-risk groups (Figures 7(c–e)). The difference in sensitivity of these compounds between high- and low-risk groups may provide new potential treatments for patients with osteosarcoma in the future.

### 3.10. GSEA and GSVA Analysis

GSEA and GSVA enrichment analysis were applied to evaluate the relationship between risk and functional pathway. GSEA analysis showed that Phenylalanine metabolism, Ribosome, and Oxidative phosphorylation were enriched in the high-risk group (Figure 7(f)); cytokine-cytokine receptor interaction, calcium signaling pathway, neuroactive ligand receptor interaction, complement and coagulation cascades, and hematopoietic cell lineage were enriched in the low-risk group (Figure 7(h)). GSVA analysis displayed that there was a close relationship between PPAR signaling pathway, neurotrophin signaling pathway, MAPK signaling pathway, calcium signaling pathway, adipocytokine signaling pathway, and risk score (Figure 7(g)). The results showed that there was a certain degree of correlation between risk score and cellular energy metabolism, tumour related pathways. These functions may be related to the progression of osteosarcoma.

### 3.11. Tumour Immune Analysis

**Figure 8:**
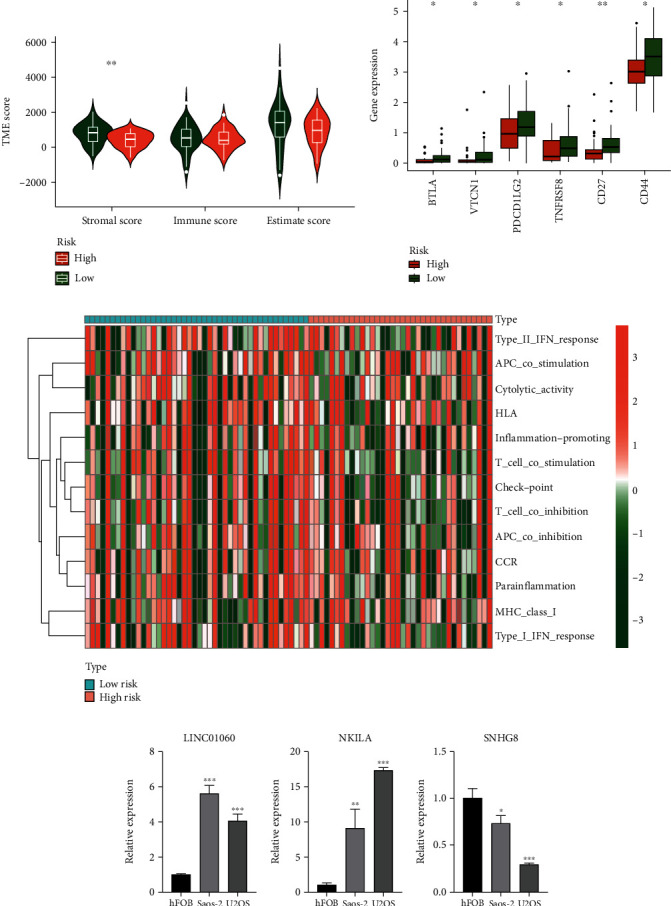
Immune correlation analysis and RT-qPCR. (a) Difference of tumour microenvironment between high- and low-risk groups, (b) Difference of expression level of immune checkpoint between high- and low-risk groups, (c) Enrichment differences of different immune functions between high- and low-risk groups, (d and e) Bar chart of RT-qPCR results (mean ± SD) (^∗^ if *P* < 0.05, ^∗∗^ if *P* < 0.01, and ^∗∗∗^ if *P* < 0.001).

The difference in tumour purity between two risk groups can be estimated by comparing the immune scores between two risk groups. It is worth noting that the analysis indicated that there was a distinction in immune score between the two risk groups, and there was a significant difference in stromal score, but on the whole, the difference was not statistically significant (Figure 8(a)). At the expression level of immune checkpoint, there were differences in the expression levels of six immune checkpoints (BTLA, VTCN1, PDCD1LG2, TNFRSF8, CD27, and CD44) between the two groups, and the expression level of six immune checkpoints in the high-risk group was lower than that in the low-risk group (Figure 8(b)). On the difference of immune function enrichment between the two risk groups, it was confirmed that there was no significant difference between the two risk groups (Figure 8(c)). These results suggested that there seems to be no difference in tumour immunity between the two risk groups.

### 3.12. Analysis of Quantitative Real-Time Polymerase Chain Reaction

Three cuproptosis-related lncRNAs (LINC01060, NKILA, and SNHG8) were chosen. hFOB, Saos-2, and U2OS cells were used to examine expression levels of these lncRNAs. The experimental results confirm our bioinformatics analysis (Figures 8(d–f)). And the result of lncRNA expression levels validated the accuracy of our research again.

## 4. Discussion

According to previous studies, it is generally believed that the mechanisms of programmed cell death include apoptosis [27], pyroptosis [28], necroptosis [29], and ferroptosis [30]. However, cuproptosis is a new method of cell death that has never been discovered previously, and its mechanism may be used to treat cancer in the future. The abundance of FDX1 and adipoacylated protein is highly correlated in various human tumours, and cell lines with high levels of fatty acylated protein are sensitive to cell death induced by copper [8, 9]. This indicates that copper ion carrier therapy should target cells with this metabolic characteristic [7]. The copper ion carrier elesclomol has been used in human clinical trials for the treatment of epithelial cancer [31, 32]. Some studies have shown that copper-containing complexes may be more effective in the treatment of osteosarcoma [33–35]. This may suggest that copper-induced death may be of great significance in the treatment of osteosarcoma. Therefore, it is worth exploring the effect of cuproptosis on tumours.

Osteosarcoma is a common malignant tumour in adolescents. There are still many problems that need to be solved in the diagnosis and treatment of osteosarcoma. In recent years, with intensifying research, many lncRNAs have been suggested to be involved in the regulation of osteosarcoma. lncRNA DARS-AS1 promotes the progression of osteosarcoma by regulating miR-532-3p/CCR7 [36]; lncRNA BACE1-AS regulates the proliferation, migration, and invasion of osteosarcoma cells through the miR-762/SOX7 axis [37]; lncRNA SNHG1 promotes osteosarcoma progression by upregulating S100A6 through miR-493-5p [38]. Some studies have shown that lncRNAs may provide clinical guidance for the treatment of osteosarcoma in the future. The study of Lee et al. confirmed that lncRNA ANRIL can be used as a biomarker of chemosensitivity and prognosis of osteosarcoma, and downregulating the expression of ANRIL may be a therapeutic strategy to overcome the current standard treatment resistance [39]. Meta-analysis of Deng et al. showed that lncRNA-XIST can be used as a potential biomarker for clinical parameters of advanced human cancer [40]. The studies of Guo et al. have shown that DSF/Cu complex can induce apoptosis and inhibit tumor progression in osteosarcoma [41]. Cheng et al. found that the chemotherapy resistance of osteosarcoma to cisplatin was changed by affecting copper transporter [42]. The research of Mandell et al. shows that mouse OS cell lines with different metastatic potential also have different levels and regulation of endogenous copper, which may contribute to the selective cytotoxicity of very low dose of copper-enhanced disulfide to K12 cells [43]. Therefore, combining the latest cuproptosis-related lncRNA risk model to predict the prognosis of patients with osteosarcoma is worth exploring. This means that the cuproptosis-related lncRNA prognostic model may be of great significance for the diagnosis and treatment of osteosarcoma. It may provide a new method for clinical diagnosis and treatment in the future.

From the TCGA database and UCSC Xena website, we acquired the expression and clinical data of osteosarcoma. 47 prognostic cuproptosis-related lncRNAs were obtained. A risk model of 9 lncRNAs was constructed by LASSO regression, and the relationship between prognosis and risk score showed that, with the decline in risk score, the prognosis of patients gradually improved. The reliability of the model was verified by a multi-index ROC curve, PCA curve, c-index curve, and comparison with other models. This suggests that the high-risk score is associated with the poor prognosis of patients. The risk model we constructed has good credibility and may be beneficial for the diagnosis and treatment of osteosarcoma in the future. By comparing the differences in IC50 between the two groups, we screened three compounds that may be beneficial for the treatment of osteosarcoma in the future. The expression levels of lncRNA were verified by RT-qPCR. In summary, these conclusions may provide a new reference for the treatment of osteosarcoma patients in the future.

This was the first study to construct a cuproptosis-related lncRNA model to predict the prognosis of patients with osteosarcoma, which has never been previously reported. Our model can effectively predict the prognosis of patients with osteosarcoma, and potential therapeutic compounds may also be beneficial for the diagnosis and treatment of osteosarcoma in the future. However, there were also some limitations in this research. First, there was a single data source for osteosarcoma. In fact, we carefully searched the major public databases, but there were too few osteosarcoma samples with complete lncRNA expression and clinical data. Therefore, more and richer osteosarcoma data are needed for our future research. Second, our research lacked advanced experimental verification, which may have made our conclusions less reliable. This is another problem that we hope to solve in the future.

## 5. Conclusion

Cuproptosis-related lncRNAs are closely related to osteosarcoma patients. Nine lncRNAs models can effectively predict the prognosis of osteosarcoma and may play an important role in individualized treatment of osteosarcoma patients in the future.

## Data Availability

All data sources and processing come from the Cancer Genome Atlas (TCGA) (https://portal.gdc.cancer.gov/repository), UCSC xena website (http://xena.ucsc.edu/), and R software (version 4.1.2.). The KEGG dataset was obtained from the GSEA website (http://www.gsea-msigdb.org/gsea/).

## Supplementary Materials

Supplementary Materials (1) Cuproptosis-related genes. (2) lncRNA corResult. (3) GO. 4. KEGG. (5) uniCox. (6) Model geneCoef. (7) GSEA Result. (8) RT-qPCR
