# Supplementary material for: The Cuproptosis-Related Long Noncoding RNA Signature Predicts Prognosis and Tumour Immune Analysis in Osteosarcoma
Source: Comput Math Methods Med. 2022 Nov 2;2022:6314182. doi: 10.1155/2022/6314182 (PMC9646308; doi:10.1155/2022/6314182)
Supplement: Supplementary Materials — (1) Cuproptosis-related genes. (2) lncRNA corResult. (3) GO. 4. KEGG. (5) uniCox. (6) Model geneCoef. (7) GSEA Result. (8) RT-qPCR [file 6314182.f1.docx]

1. Cuproptosis-related genes

| Gene |
| --- |
| NFE2L2 |
| NLRP3 |
| ATP7B |
| ATP7A |
| SLC31A1 |
| FDX1 |
| LIAS |
| LIPT1 |
| LIPT2 |
| DLD |
| DLAT |
| PDHA1 |
| PDHB |
| MTF1 |
| GLS |
| CDKN2A |
| DBT |
| GCSH |
| DLST |

2. lncRNA corResult

| cuproptosis | lncRNA | cor | pvalue | Regulation |
| --- | --- | --- | --- | --- |
| DLD | AC139792.1 | 0.469288 | 7.63E-06 | postive |
| MTF1 | SRGAP2-AS1 | 0.478216 | 4.82E-06 | postive |
| FDX1 | AL033527.3 | 0.431095 | 4.73E-05 | postive |
| DLD | AC093297.2 | 0.401621 | 0.000168 | postive |
| GCSH | AC092611.1 | 0.416 | 9.18E-05 | postive |
| GCSH | AC139768.1 | 0.485718 | 3.25E-06 | postive |
| DLD | LINC00964 | 0.451299 | 1.85E-05 | postive |
| MTF1 | C12orf77 | 0.405952 | 0.00014 | postive |
| LIPT1 | LINC00921 | 0.410538 | 0.000116 | postive |
| NLRP3 | AC004847.1 | 0.516652 | 5.73E-07 | postive |
| CDKN2A | CDKN2A-DT | 0.513485 | 6.90E-07 | postive |
| PDHA1 | AC243829.5 | 0.408412 | 0.000127 | postive |
| DLD | AC078795.1 | 0.427221 | 5.63E-05 | postive |
| LIPT1 | AC074117.1 | 0.400003 | 0.000179 | postive |
| SLC31A1 | DNM3OS | 0.400373 | 0.000177 | postive |
| GLS | DNM3OS | 0.518875 | 5.03E-07 | postive |
| LIPT1 | AC002546.1 | 0.52009 | 4.68E-07 | postive |
| FDX1 | LINC02551 | 0.408434 | 0.000127 | postive |
| LIPT1 | AC008434.1 | 0.426901 | 5.71E-05 | postive |
| MTF1 | AC078777.1 | 0.570716 | 1.77E-08 | postive |
| PDHA1 | AL157911.1 | 0.410174 | 0.000118 | postive |
| GLS | AP001001.1 | 0.404412 | 0.00015 | postive |
| LIPT1 | AC092718.5 | 0.541993 | 1.21E-07 | postive |
| MTF1 | AL359397.1 | 0.415063 | 9.56E-05 | postive |
| PDHB | MEG8 | 0.423655 | 6.58E-05 | postive |
| LIPT1 | AP002807.1 | 0.538853 | 1.48E-07 | postive |
| ATP7A | AC104532.2 | 0.468414 | 7.98E-06 | postive |
| LIPT1 | AL133297.2 | 0.525247 | 3.43E-07 | postive |
| PDHB | MIR1-1HG-AS1 | 0.446135 | 2.37E-05 | postive |
| LIPT1 | LINC00677 | 0.694108 | 3.44E-13 | postive |
| LIPT1 | AL354928.1 | 0.513306 | 6.97E-07 | postive |
| DLD | AC131011.1 | 0.440733 | 3.05E-05 | postive |
| LIPT1 | AP001020.3 | 0.421616 | 7.20E-05 | postive |
| LIPT1 | AC107214.2 | 0.523096 | 3.91E-07 | postive |
| NFE2L2 | AL121749.1 | 0.407817 | 0.00013 | postive |
| LIPT1 | INTS6-AS1 | 0.626844 | 2.30E-10 | postive |
| DLD | AC010201.2 | 0.480298 | 4.33E-06 | postive |
| DLD | LINC02237 | 0.425403 | 6.10E-05 | postive |
| GCSH | LINC00665 | 0.452532 | 1.75E-05 | postive |
| DLD | LINC00603 | 0.484892 | 3.39E-06 | postive |
| DLD | AC084083.1 | 0.454245 | 1.61E-05 | postive |
| FDX1 | LINC01836 | 0.475392 | 5.58E-06 | postive |
| LIAS | AC025162.2 | 0.439398 | 3.24E-05 | postive |
| LIPT1 | AC025162.2 | 0.654455 | 1.94E-11 | postive |
| LIPT1 | LINC00294 | 0.40106 | 0.000172 | postive |
| LIPT1 | HIPK1-AS1 | 0.494707 | 2.00E-06 | postive |
| LIPT1 | AC007686.3 | 0.467447 | 8.38E-06 | postive |
| LIPT1 | HHLA3 | 0.50784 | 9.56E-07 | postive |
| LIPT1 | LINC01569 | 0.615996 | 5.70E-10 | postive |
| DLD | AC010967.1 | 0.622111 | 3.44E-10 | postive |
| FDX1 | AC025741.1 | 0.434651 | 4.03E-05 | postive |
| LIPT1 | ARHGEF26-AS1 | 0.609739 | 9.47E-10 | postive |
| FDX1 | MESTIT1 | 0.41019 | 0.000118 | postive |
| PDHB | AL031429.2 | 0.409752 | 0.00012 | postive |
| LIPT2 | AL136040.1 | 0.476348 | 5.31E-06 | postive |
| LIPT1 | AC018690.1 | 0.586618 | 5.62E-09 | postive |
| LIPT1 | AC005730.3 | 0.50763 | 9.67E-07 | postive |
| FDX1 | AC124798.1 | 0.614945 | 6.21E-10 | postive |
| NLRP3 | AC138207.5 | 0.689934 | 5.43E-13 | postive |
| LIPT1 | ZNF213-AS1 | 0.590381 | 4.25E-09 | postive |
| GCSH | LINC01801 | 0.415213 | 9.49E-05 | postive |
| NLRP3 | AC006033.2 | 0.450488 | 1.93E-05 | postive |
| LIAS | AC022148.1 | 0.463234 | 1.03E-05 | postive |
| MTF1 | AF038458.3 | 0.600199 | 2.01E-09 | postive |
| LIPT1 | AL513218.1 | 0.570575 | 1.79E-08 | postive |
| NLRP3 | AC110995.1 | 0.665056 | 7.02E-12 | postive |
| NLRP3 | LINC01150 | 0.738672 | 1.58E-15 | postive |
| LIPT1 | AC116447.1 | 0.571114 | 1.72E-08 | postive |
| FDX1 | AP003025.1 | 0.440089 | 3.14E-05 | postive |
| MTF1 | AC024075.3 | 0.573225 | 1.48E-08 | postive |
| DBT | AC024075.3 | 0.472684 | 6.42E-06 | postive |
| ATP7A | AC009955.4 | 0.411948 | 0.000109 | postive |
| PDHB | SNHG8 | 0.428178 | 5.39E-05 | postive |
| LIPT2 | LINC01597 | 0.413093 | 0.000104 | postive |
| GCSH | AC091729.2 | 0.435827 | 3.82E-05 | postive |
| DLD | AC021739.2 | 0.493456 | 2.14E-06 | postive |
| DLST | AL137058.2 | 0.40536 | 0.000144 | postive |
| LIPT1 | AC004846.2 | 0.477492 | 5.01E-06 | postive |
| DLD | LINC01781 | 0.428486 | 5.32E-05 | postive |
| MTF1 | AC068491.2 | 0.401164 | 0.000171 | postive |
| MTF1 | AL592166.1 | 0.54908 | 7.66E-08 | postive |
| NLRP3 | PCED1B-AS1 | 0.612091 | 7.84E-10 | postive |
| MTF1 | AC008147.2 | 0.409633 | 0.00012 | postive |
| FDX1 | AC027097.1 | 0.414963 | 9.60E-05 | postive |
| FDX1 | AC006441.1 | 0.403587 | 0.000155 | postive |
| GCSH | AC125437.1 | 0.429673 | 5.04E-05 | postive |
| GCSH | AC131025.1 | 0.434614 | 4.03E-05 | postive |
| LIPT1 | AC012557.1 | 0.541893 | 1.22E-07 | postive |
| ATP7A | LINC00922 | 0.415246 | 9.48E-05 | postive |
| SLC31A1 | AC011477.2 | 0.459264 | 1.26E-05 | postive |
| DLD | AC011477.2 | 0.415804 | 9.26E-05 | postive |
| GLS | AC011477.2 | 0.542914 | 1.14E-07 | postive |
| DLD | AC063948.1 | 0.478892 | 4.66E-06 | postive |
| GLS | AC113189.3 | 0.499237 | 1.55E-06 | postive |
| NFE2L2 | FTX | 0.419679 | 7.83E-05 | postive |
| ATP7A | FTX | 0.555337 | 5.07E-08 | postive |
| GLS | FTX | 0.412643 | 0.000106 | postive |
| DLAT | BACE1-AS | 0.407554 | 0.000131 | postive |
| ATP7A | AC068790.5 | 0.444305 | 2.58E-05 | postive |
| FDX1 | AC011816.2 | 0.421294 | 7.30E-05 | postive |
| MTF1 | AC024075.1 | 0.685809 | 8.44E-13 | postive |
| DBT | AC024075.1 | 0.556533 | 4.68E-08 | postive |
| DLD | AC073534.2 | 0.504537 | 1.15E-06 | postive |
| DLD | AC010834.2 | 0.486762 | 3.07E-06 | postive |
| DLD | AC025580.1 | 0.476596 | 5.25E-06 | postive |
| DBT | AC104653.1 | 0.421784 | 7.15E-05 | postive |
| PDHA1 | AC008438.1 | 0.424363 | 6.38E-05 | postive |
| LIPT1 | LINC01013 | 0.677241 | 2.07E-12 | postive |
| FDX1 | AC005277.2 | 0.463154 | 1.04E-05 | postive |
| MTF1 | AC079760.1 | 0.400543 | 0.000175 | postive |
| GCSH | AL450344.2 | 0.476131 | 5.37E-06 | postive |
| LIAS | WARS2-AS1 | 0.435132 | 3.94E-05 | postive |
| LIPT1 | WARS2-AS1 | 0.551179 | 6.68E-08 | postive |
| LIPT2 | AC005076.1 | 0.412966 | 0.000104 | postive |
| FDX1 | AP002884.3 | 0.414302 | 9.87E-05 | postive |
| LIPT1 | AC024337.2 | 0.472686 | 6.42E-06 | postive |
| GLS | AC005104.1 | 0.422811 | 6.83E-05 | postive |
| MTF1 | AC010273.2 | 0.483693 | 3.62E-06 | postive |
| DBT | AC010273.2 | 0.429082 | 5.18E-05 | postive |
| LIPT1 | AC136424.2 | 0.600577 | 1.95E-09 | postive |
| LIPT1 | ZNF528-AS1 | 0.536913 | 1.67E-07 | postive |
| LIAS | AC092718.3 | 0.419168 | 8.01E-05 | postive |
| LIPT1 | AC092718.3 | 0.477325 | 5.05E-06 | postive |
| DLD | AC022872.1 | 0.454696 | 1.57E-05 | postive |
| LIPT2 | AP002360.1 | 0.408411 | 0.000127 | postive |
| MTF1 | FARSA-AS1 | 0.4302 | 4.92E-05 | postive |
| DBT | FARSA-AS1 | 0.407928 | 0.000129 | postive |
| MTF1 | AC022400.2 | 0.40693 | 0.000135 | postive |
| MTF1 | AL590093.1 | 0.40829 | 0.000127 | postive |
| DLD | AP001043.1 | 0.421913 | 7.11E-05 | postive |
| MTF1 | AC009690.2 | 0.421451 | 7.25E-05 | postive |
| PDHA1 | AC009063.3 | 0.411003 | 0.000114 | postive |
| FDX1 | AC105254.1 | 0.415071 | 9.55E-05 | postive |
| LIPT1 | AC006141.1 | 0.540084 | 1.37E-07 | postive |
| MTF1 | SLC2A1-AS1 | 0.469643 | 7.50E-06 | postive |
| DLST | AC005225.3 | 0.427772 | 5.49E-05 | postive |
| LIAS | AC125807.2 | 0.418955 | 8.08E-05 | postive |
| DLD | RSF1-IT2 | 0.460516 | 1.18E-05 | postive |
| DLD | AC103691.1 | 0.490312 | 2.54E-06 | postive |
| NFE2L2 | EIPR1-IT1 | 0.401901 | 0.000166 | postive |
| LIPT2 | AL135744.1 | 0.40633 | 0.000138 | postive |
| MTF1 | AC002558.3 | 0.438095 | 3.44E-05 | postive |
| FDX1 | AC020663.3 | 0.461755 | 1.11E-05 | postive |
| LIPT1 | ARIH2OS | 0.46025 | 1.20E-05 | postive |
| GCSH | AC093484.4 | 0.535972 | 1.77E-07 | postive |
| LIPT1 | AC145285.3 | 0.550055 | 7.19E-08 | postive |
| GCSH | AC002116.2 | 0.475107 | 5.67E-06 | postive |
| NLRP3 | AC018755.4 | 0.475884 | 5.44E-06 | postive |
| PDHA1 | AL118505.1 | 0.464832 | 9.55E-06 | postive |
| LIPT1 | AL031058.1 | 0.410611 | 0.000115 | postive |
| ATP7A | LINC02392 | 0.405563 | 0.000143 | postive |
| PDHB | AL512625.2 | 0.406596 | 0.000137 | postive |
| LIPT1 | AL354892.3 | 0.566804 | 2.32E-08 | postive |
| DLD | AL591721.1 | 0.416724 | 8.90E-05 | postive |
| NFE2L2 | LINC01213 | 0.432641 | 4.41E-05 | postive |
| DLST | AL157871.5 | 0.40744 | 0.000132 | postive |
| DLD | AC009831.3 | 0.441306 | 2.97E-05 | postive |
| ATP7A | SNHG11 | -0.46195 | 1.10E-05 | negative |
| GCSH | AC092718.4 | 0.40719 | 0.000133 | postive |
| DLD | AL442163.1 | 0.408094 | 0.000128 | postive |
| DLST | DNAJC9-AS1 | 0.412521 | 0.000106 | postive |
| DLD | LINC01443 | 0.493072 | 2.18E-06 | postive |
| PDHA1 | AC023194.2 | 0.409515 | 0.000121 | postive |
| PDHA1 | AC087721.1 | 0.408675 | 0.000125 | postive |
| FDX1 | LINC01060 | 0.536752 | 1.69E-07 | postive |
| FDX1 | AC009495.3 | 0.423764 | 6.55E-05 | postive |
| FDX1 | AP000851.2 | 0.590167 | 4.32E-09 | postive |
| SLC31A1 | EBLN3P | 0.451733 | 1.81E-05 | postive |
| GLS | EBLN3P | 0.443244 | 2.71E-05 | postive |
| SLC31A1 | LINC01775 | 0.440465 | 3.08E-05 | postive |
| DLD | AL359317.2 | 0.527186 | 3.05E-07 | postive |
| LIPT1 | KCNIP2-AS1 | 0.480506 | 4.28E-06 | postive |
| DLD | AP003354.1 | 0.445347 | 2.46E-05 | postive |
| FDX1 | LINC00837 | 0.480754 | 4.22E-06 | postive |
| DLD | AL513318.1 | 0.407946 | 0.000129 | postive |
| PDHB | AL513318.1 | 0.506003 | 1.06E-06 | postive |
| PDHB | AC040160.1 | -0.40614 | 0.000139 | negative |
| PDHB | DUBR | 0.45477 | 1.57E-05 | postive |
| GCSH | AC008393.1 | 0.419033 | 8.06E-05 | postive |
| DLD | AP002340.1 | 0.419424 | 7.92E-05 | postive |
| MTF1 | AL390728.4 | -0.41018 | 0.000118 | negative |
| MTF1 | AP005131.5 | 0.4693 | 7.63E-06 | postive |
| MTF1 | LINC00963 | 0.467198 | 8.48E-06 | postive |
| LIPT1 | AC016735.1 | 0.427037 | 5.67E-05 | postive |
| DBT | AC105206.2 | 0.406663 | 0.000136 | postive |
| GLS | AL049869.2 | 0.503756 | 1.21E-06 | postive |
| DLAT | AL035071.2 | 0.441927 | 2.88E-05 | postive |
| DLD | AC004039.1 | 0.44419 | 2.59E-05 | postive |
| FDX1 | ATP2A1-AS1 | 0.42752 | 5.55E-05 | postive |
| LIAS | FAM27E3 | 0.404099 | 0.000151 | postive |
| LIPT1 | FAM27E3 | 0.45638 | 1.45E-05 | postive |
| MTF1 | AC104109.4 | 0.443061 | 2.73E-05 | postive |
| LIAS | AC107398.2 | 0.407023 | 0.000134 | postive |
| LIPT1 | AC107398.2 | 0.617962 | 4.85E-10 | postive |
| DLD | PSMD6-AS2 | 0.484228 | 3.52E-06 | postive |
| NLRP3 | AL133371.2 | 0.538503 | 1.51E-07 | postive |
| DLD | AL157400.4 | 0.409246 | 0.000122 | postive |
| DLD | SNHG6 | 0.412448 | 0.000107 | postive |
| PDHB | SNHG6 | 0.458806 | 1.29E-05 | postive |
| FDX1 | LINC01923 | 0.42599 | 5.94E-05 | postive |
| DLST | AL356966.1 | 0.434873 | 3.99E-05 | postive |
| MTF1 | AC005070.3 | 0.416674 | 8.92E-05 | postive |
| LIPT1 | NSMCE1-DT | 0.43824 | 3.42E-05 | postive |
| PDHB | AC003965.1 | 0.430663 | 4.82E-05 | postive |
| GLS | AC010655.2 | 0.409687 | 0.00012 | postive |
| PDHB | AC115837.1 | 0.405126 | 0.000145 | postive |
| LIPT2 | NFE4 | 0.411181 | 0.000113 | postive |
| FDX1 | AC020911.2 | 0.438958 | 3.31E-05 | postive |
| GCSH | ERVK-28 | 0.448053 | 2.16E-05 | postive |
| DLD | AC116345.1 | 0.41467 | 9.72E-05 | postive |
| LIAS | AC004943.2 | 0.42092 | 7.42E-05 | postive |
| MTF1 | PRORY | 0.517735 | 5.38E-07 | postive |
| FDX1 | BMS1P4 | 0.412012 | 0.000109 | postive |
| LIPT1 | BX571846.1 | 0.528413 | 2.83E-07 | postive |
| NFE2L2 | AC007365.1 | 0.473128 | 6.27E-06 | postive |
| LIPT1 | AC114810.1 | 0.402239 | 0.000164 | postive |
| LIPT1 | AC097641.1 | 0.594784 | 3.05E-09 | postive |
| LIPT1 | AC027801.1 | 0.422378 | 6.96E-05 | postive |
| GCSH | AC090517.2 | 0.404959 | 0.000146 | postive |
| GCSH | BDNF-AS | 0.42764 | 5.52E-05 | postive |
| GCSH | AC009107.2 | 0.458651 | 1.30E-05 | postive |
| LIPT1 | AC093525.6 | 0.445141 | 2.48E-05 | postive |
| MTF1 | AL157935.1 | 0.501387 | 1.38E-06 | postive |
| ATP7A | AC099811.1 | 0.487659 | 2.93E-06 | postive |
| MTF1 | AC005224.2 | 0.422376 | 6.96E-05 | postive |
| PDHB | AC007279.1 | 0.456007 | 1.48E-05 | postive |
| LIAS | RNASEH1-AS1 | 0.458617 | 1.30E-05 | postive |
| LIPT2 | RNASEH1-AS1 | 0.409415 | 0.000121 | postive |
| LIPT2 | AC100812.1 | 0.411291 | 0.000112 | postive |
| ATP7A | LINC00629 | 0.405573 | 0.000143 | postive |
| GCSH | AC010632.2 | 0.5378 | 1.58E-07 | postive |
| MTF1 | Z97200.1 | 0.609318 | 9.79E-10 | postive |
| FDX1 | AC037198.1 | 0.490666 | 2.49E-06 | postive |
| ATP7A | Z68871.1 | 0.404179 | 0.000151 | postive |
| MTF1 | BEAN1-AS1 | 0.420706 | 7.49E-05 | postive |
| LIPT1 | AP005131.3 | 0.615924 | 5.74E-10 | postive |
| DLST | AL356481.3 | 0.413036 | 0.000104 | postive |
| MTF1 | AC010300.1 | 0.494852 | 1.98E-06 | postive |
| DBT | AC010300.1 | 0.431891 | 4.56E-05 | postive |
| MTF1 | NGF-AS1 | 0.466211 | 8.91E-06 | postive |
| DBT | NGF-AS1 | 0.41193 | 0.000109 | postive |
| LIPT1 | AC007546.1 | 0.417115 | 8.75E-05 | postive |
| GCSH | AC108727.1 | 0.419754 | 7.81E-05 | postive |
| SLC31A1 | FAM225B | 0.439626 | 3.21E-05 | postive |
| MTF1 | AC087392.1 | 0.413431 | 0.000102 | postive |
| LIPT2 | AC008026.3 | -0.40985 | 0.000119 | negative |
| NLRP3 | AC138207.1 | 0.52231 | 4.09E-07 | postive |
| NLRP3 | AC138207.4 | 0.68321 | 1.11E-12 | postive |
| GCSH | AC135803.1 | 0.459821 | 1.22E-05 | postive |
| NLRP3 | AL357033.4 | 0.436089 | 3.77E-05 | postive |
| GLS | FARP1-AS1 | 0.436475 | 3.71E-05 | postive |
| DBT | LINC00205 | 0.416456 | 9.00E-05 | postive |
| GCSH | LINC00173 | 0.505295 | 1.11E-06 | postive |
| NFE2L2 | AC114760.2 | 0.531974 | 2.27E-07 | postive |
| GLS | AC114760.2 | 0.413804 | 0.000101 | postive |
| ATP7A | AC005746.1 | 0.406842 | 0.000135 | postive |
| GCSH | AC005746.1 | 0.403239 | 0.000157 | postive |
| MTF1 | NNT-AS1 | 0.549351 | 7.53E-08 | postive |
| DBT | NNT-AS1 | 0.467322 | 8.43E-06 | postive |
| ATP7B | DLEU2 | 0.42077 | 7.47E-05 | postive |
| GCSH | AC084116.3 | 0.46043 | 1.19E-05 | postive |
| DLAT | AC127537.1 | -0.42018 | 7.66E-05 | negative |
| DLD | AC107871.2 | 0.475102 | 5.67E-06 | postive |
| DLD | AC087071.2 | 0.433522 | 4.24E-05 | postive |
| NLRP3 | LINC02285 | 0.425535 | 6.06E-05 | postive |
| DLD | AL162311.1 | 0.40825 | 0.000127 | postive |
| LIPT1 | AC091588.3 | 0.586723 | 5.58E-09 | postive |
| LIPT1 | AC011498.1 | 0.635853 | 1.06E-10 | postive |
| LIPT1 | AC018521.5 | 0.61231 | 7.70E-10 | postive |
| MTF1 | AL807757.2 | 0.49442 | 2.03E-06 | postive |
| CDKN2A | AC137894.1 | 0.41472 | 9.70E-05 | postive |
| DLAT | NBAT1 | 0.405248 | 0.000144 | postive |
| ATP7B | AL117209.1 | 0.444503 | 2.56E-05 | postive |
| PDHB | GLIDR | 0.448696 | 2.10E-05 | postive |
| LIPT1 | AL139246.3 | 0.45851 | 1.31E-05 | postive |
| DLD | AC100858.2 | 0.458862 | 1.28E-05 | postive |
| LIPT1 | AC009812.4 | 0.641274 | 6.53E-11 | postive |
| PDHA1 | LINC02308 | 0.441221 | 2.98E-05 | postive |
| LIAS | AL391834.2 | 0.42103 | 7.39E-05 | postive |
| LIPT1 | AL391834.2 | 0.404282 | 0.00015 | postive |
| ATP7A | AL031118.1 | 0.433424 | 4.26E-05 | postive |
| GCSH | AC079414.3 | 0.408823 | 0.000124 | postive |
| GLS | AC091849.2 | 0.434298 | 4.09E-05 | postive |
| PDHA1 | AC119800.1 | 0.455744 | 1.49E-05 | postive |
| DLST | AC119800.1 | 0.404542 | 0.000149 | postive |
| DLST | ZNF710-AS1 | 0.440814 | 3.04E-05 | postive |
| LIAS | AC092809.2 | -0.40601 | 0.00014 | negative |
| GCSH | AC007570.1 | 0.428661 | 5.28E-05 | postive |
| NLRP3 | IL10RB-DT | 0.410889 | 0.000114 | postive |
| MTF1 | AC018462.1 | 0.452946 | 1.71E-05 | postive |
| DBT | AC018462.1 | 0.410006 | 0.000118 | postive |
| MTF1 | AP001099.1 | 0.487686 | 2.92E-06 | postive |
| LIPT1 | AP001922.6 | 0.449452 | 2.02E-05 | postive |
| GCSH | AC087362.1 | 0.40328 | 0.000157 | postive |
| LIPT1 | AC018450.1 | 0.488829 | 2.75E-06 | postive |
| CDKN2A | AL121832.3 | 0.47549 | 5.56E-06 | postive |
| ATP7A | LINC00630 | 0.452501 | 1.75E-05 | postive |
| PDHB | LINC02610 | 0.485989 | 3.20E-06 | postive |
| DLD | GHET1 | 0.53718 | 1.64E-07 | postive |
| PDHA1 | AC006160.1 | 0.423361 | 6.67E-05 | postive |
| PDHA1 | ATXN2-AS | 0.400947 | 0.000172 | postive |
| DLAT | SMARCA5-AS1 | 0.40115 | 0.000171 | postive |
| DLAT | AL031123.2 | 0.402629 | 0.000161 | postive |
| PDHB | AL031123.2 | 0.509734 | 8.57E-07 | postive |
| LIPT1 | AC091185.1 | 0.527076 | 3.07E-07 | postive |
| GCSH | AL731567.1 | 0.405486 | 0.000143 | postive |
| LIPT2 | LINC01106 | 0.497657 | 1.70E-06 | postive |
| ATP7A | AC068790.4 | 0.473364 | 6.20E-06 | postive |
| GCSH | AC091045.1 | 0.406584 | 0.000137 | postive |
| GLS | LINC02242 | 0.456768 | 1.42E-05 | postive |
| SLC31A1 | AL512785.1 | 0.40125 | 0.00017 | postive |
| LIPT2 | AL512785.1 | 0.410794 | 0.000115 | postive |
| GLS | AL512785.1 | 0.509461 | 8.71E-07 | postive |
| LIPT1 | LINC02204 | 0.5062 | 1.05E-06 | postive |
| MTF1 | AL031283.2 | 0.464676 | 9.63E-06 | postive |
| PDHB | AL121655.1 | 0.437251 | 3.58E-05 | postive |
| PDHB | AC069222.1 | 0.419294 | 7.96E-05 | postive |
| NFE2L2 | AC005021.1 | 0.401569 | 0.000168 | postive |
| ATP7B | AC005021.1 | 0.415141 | 9.52E-05 | postive |
| PDHA1 | AC080129.2 | 0.455002 | 1.55E-05 | postive |
| MTF1 | AP000962.1 | 0.477436 | 5.02E-06 | postive |
| GLS | LINC00640 | 0.444648 | 2.54E-05 | postive |
| LIPT1 | AC090192.2 | 0.579028 | 9.79E-09 | postive |
| GCSH | TMEM147-AS1 | 0.42645 | 5.82E-05 | postive |
| MTF1 | AC060766.6 | 0.459186 | 1.26E-05 | postive |
| PDHA1 | AL023806.1 | 0.484044 | 3.55E-06 | postive |
| MTF1 | AL021937.4 | 0.436433 | 3.71E-05 | postive |
| LIPT1 | LINC02609 | 0.402903 | 0.000159 | postive |
| GCSH | AC064836.3 | 0.471499 | 6.82E-06 | postive |
| DLD | Z99758.1 | 0.477954 | 4.89E-06 | postive |
| DLD | RPARP-AS1 | 0.441973 | 2.88E-05 | postive |
| MTF1 | AC027698.1 | 0.481051 | 4.16E-06 | postive |
| PDHA1 | HIF1A-AS1 | 0.417464 | 8.62E-05 | postive |
| DLST | HIF1A-AS1 | 0.4176 | 8.57E-05 | postive |
| LIPT2 | AP001372.2 | 0.471535 | 6.81E-06 | postive |
| GLS | AL513534.2 | 0.483072 | 3.74E-06 | postive |
| GCSH | AL392023.2 | 0.40746 | 0.000132 | postive |
| PDHA1 | AC004264.1 | 0.401973 | 0.000165 | postive |
| DLD | AP003065.1 | 0.449607 | 2.01E-05 | postive |
| LIPT1 | AC002511.2 | 0.656746 | 1.57E-11 | postive |
| MTF1 | AC092683.1 | 0.409719 | 0.00012 | postive |
| LIAS | AL590705.3 | 0.414821 | 9.66E-05 | postive |
| LIPT1 | AL590705.3 | 0.681819 | 1.29E-12 | postive |
| FDX1 | AC090152.1 | -0.40577 | 0.000141 | negative |
| FDX1 | AC100821.2 | 0.437542 | 3.53E-05 | postive |
| DLD | LINC02255 | 0.409853 | 0.000119 | postive |
| LIPT1 | LINC01960 | 0.675805 | 2.39E-12 | postive |
| GCSH | AC024270.1 | 0.537661 | 1.59E-07 | postive |
| PDHB | AC009812.1 | 0.418443 | 8.26E-05 | postive |
| MTF1 | AC068481.1 | 0.583063 | 7.30E-09 | postive |
| LIPT1 | AC105384.1 | 0.668251 | 5.12E-12 | postive |
| DLST | AC004825.2 | 0.450316 | 1.94E-05 | postive |
| NFE2L2 | LINC01614 | 0.429288 | 5.13E-05 | postive |
| FDX1 | AL391422.4 | -0.4025 | 0.000162 | negative |
| GLS | AP001432.1 | 0.475136 | 5.66E-06 | postive |
| GLS | AC084782.2 | 0.440574 | 3.07E-05 | postive |
| GCSH | AC084757.4 | 0.413744 | 0.000101 | postive |
| MTF1 | HEXD-IT1 | 0.416776 | 8.88E-05 | postive |
| LIPT1 | AC106820.2 | 0.441326 | 2.96E-05 | postive |
| ATP7A | PAXIP1-AS2 | 0.427509 | 5.55E-05 | postive |
| LIPT1 | AC145423.1 | 0.522344 | 4.09E-07 | postive |
| GCSH | AC004241.3 | 0.411306 | 0.000112 | postive |
| GCSH | MAGI1-IT1 | 0.415439 | 9.40E-05 | postive |
| LIPT1 | AC005740.4 | 0.418432 | 8.27E-05 | postive |
| PDHA1 | AC005597.1 | 0.429298 | 5.13E-05 | postive |
| LIAS | AL359504.1 | 0.410205 | 0.000117 | postive |
| LIPT1 | AL359504.1 | 0.564266 | 2.77E-08 | postive |
| GCSH | AC087588.1 | 0.415804 | 9.26E-05 | postive |
| DLD | AC015722.2 | 0.405939 | 0.00014 | postive |
| FDX1 | AC021242.3 | 0.426288 | 5.86E-05 | postive |
| DLD | AC027018.1 | 0.452717 | 1.73E-05 | postive |
| GCSH | AC092296.2 | 0.43481 | 4.00E-05 | postive |
| DLD | AC004492.1 | 0.451189 | 1.86E-05 | postive |
| DLD | LINC00668 | 0.419184 | 8.00E-05 | postive |
| PDHB | LINC00668 | 0.459638 | 1.24E-05 | postive |
| LIPT1 | AL355385.1 | 0.40166 | 0.000167 | postive |
| PDHB | SNHG14 | 0.411542 | 0.000111 | postive |
| MTF1 | AC087257.1 | 0.512684 | 7.23E-07 | postive |
| GCSH | AC044802.1 | 0.423126 | 6.74E-05 | postive |
| ATP7B | AC107023.1 | 0.424846 | 6.25E-05 | postive |
| MTF1 | AC024075.2 | 0.494972 | 1.97E-06 | postive |
| DBT | AC024075.2 | 0.416809 | 8.87E-05 | postive |
| GLS | AC096586.2 | 0.444763 | 2.52E-05 | postive |
| CDKN2A | AL139423.1 | 0.427629 | 5.52E-05 | postive |
| LIPT1 | AC079313.2 | 0.420087 | 7.70E-05 | postive |
| DLAT | AC004908.1 | 0.47607 | 5.39E-06 | postive |
| MTF1 | AC005776.2 | 0.616875 | 5.31E-10 | postive |
| LIPT1 | DM1-AS | 0.434455 | 4.06E-05 | postive |
| NFE2L2 | LINC02544 | 0.402575 | 0.000161 | postive |
| DBT | AC064807.4 | 0.404744 | 0.000148 | postive |
| NLRP3 | AC243960.1 | 0.510001 | 8.44E-07 | postive |
| NLRP3 | AL161785.1 | 0.682441 | 1.20E-12 | postive |
| LIPT1 | AC087164.1 | 0.482812 | 3.79E-06 | postive |
| LIPT1 | AC005479.2 | 0.420712 | 7.49E-05 | postive |
| GCSH | AC008870.4 | 0.418828 | 8.13E-05 | postive |
| ATP7A | Z83843.1 | 0.45714 | 1.40E-05 | postive |
| GLS | Z83843.1 | 0.4651 | 9.42E-06 | postive |
| PDHA1 | AC069307.1 | 0.419451 | 7.91E-05 | postive |
| DLST | AC069307.1 | 0.411632 | 0.000111 | postive |
| MTF1 | AC126773.4 | 0.475139 | 5.66E-06 | postive |
| NLRP3 | AC113143.1 | 0.409492 | 0.000121 | postive |
| LIPT2 | AC002467.1 | 0.512128 | 7.47E-07 | postive |
| ATP7A | AP001107.5 | 0.455835 | 1.49E-05 | postive |
| DLD | AL353771.1 | 0.414743 | 9.69E-05 | postive |
| FDX1 | NKILA | -0.4858 | 3.23E-06 | negative |
| NLRP3 | AC007342.1 | 0.400864 | 0.000173 | postive |
| ATP7B | AL139241.1 | 0.436083 | 3.77E-05 | postive |
| DLD | AC018742.1 | 0.418364 | 8.29E-05 | postive |
| GCSH | AL451050.2 | 0.479284 | 4.56E-06 | postive |
| PDHA1 | AL356108.1 | 0.429889 | 4.99E-05 | postive |
| LIPT1 | AL365295.1 | 0.540019 | 1.37E-07 | postive |
| LIPT1 | AL136295.2 | 0.509324 | 8.78E-07 | postive |
| DLD | AP002336.2 | 0.473671 | 6.10E-06 | postive |
| NLRP3 | LINC01094 | 0.592974 | 3.49E-09 | postive |
| NLRP3 | HLA-DQB1-AS1 | 0.547236 | 8.64E-08 | postive |
| LIPT2 | LINC00601 | 0.41663 | 8.94E-05 | postive |
| GCSH | AC080013.2 | 0.408166 | 0.000128 | postive |
| GCSH | LINC00240 | 0.452025 | 1.79E-05 | postive |
| ATP7A | AC234775.2 | 0.43264 | 4.41E-05 | postive |
| SLC31A1 | AC234775.2 | 0.411215 | 0.000113 | postive |
| MTF1 | MIR99AHG | 0.525978 | 3.28E-07 | postive |
| PDHB | AC090204.1 | 0.425152 | 6.16E-05 | postive |
| LIPT1 | AC009509.4 | 0.518935 | 5.01E-07 | postive |
| MTF1 | AC027277.2 | 0.540365 | 1.34E-07 | postive |
| FDX1 | LINC01433 | 0.54107 | 1.28E-07 | postive |
| NFE2L2 | AC109587.1 | 0.45741 | 1.38E-05 | postive |
| DLD | LINC01695 | 0.404955 | 0.000146 | postive |
| LIPT1 | TAPT1-AS1 | 0.473984 | 6.00E-06 | postive |
| LIPT1 | SNTG2-AS1 | 0.452412 | 1.76E-05 | postive |
| PDHA1 | LINC01560 | 0.434105 | 4.13E-05 | postive |
| PDHB | AC034236.2 | 0.445652 | 2.42E-05 | postive |
| MTF1 | SAP30L-AS1 | 0.472587 | 6.45E-06 | postive |
| LIPT1 | FMR1-IT1 | 0.531246 | 2.38E-07 | postive |
| DBT | BACH1-AS1 | 0.401847 | 0.000166 | postive |
| NLRP3 | AC090559.1 | 0.670031 | 4.29E-12 | postive |
| MTF1 | BACH1-IT3 | 0.439481 | 3.23E-05 | postive |
| DLST | LINC01423 | 0.41609 | 9.14E-05 | postive |
| GCSH | AC026333.4 | 0.503452 | 1.23E-06 | postive |
| MTF1 | GASAL1 | 0.417651 | 8.55E-05 | postive |
| MTF1 | AC069549.1 | 0.425264 | 6.13E-05 | postive |
| NLRP3 | CARD8-AS1 | 0.561117 | 3.43E-08 | postive |
| LIPT2 | AC007384.1 | 0.410134 | 0.000118 | postive |
| ATP7A | AL355075.2 | 0.44473 | 2.53E-05 | postive |
| LIPT1 | LINC02615 | 0.406373 | 0.000138 | postive |
| MTF1 | UBE2R2-AS1 | 0.420029 | 7.71E-05 | postive |
| MTF1 | AC005086.2 | 0.417687 | 8.54E-05 | postive |
| MTF1 | HDHD5-AS1 | 0.408192 | 0.000128 | postive |
| GCSH | BX890604.1 | 0.554123 | 5.50E-08 | postive |
| LIPT1 | AC017048.3 | 0.591503 | 3.90E-09 | postive |
| MTF1 | AL162171.2 | 0.483601 | 3.63E-06 | postive |
| GCSH | AC079610.1 | 0.43632 | 3.73E-05 | postive |
| NLRP3 | MACORIS | 0.413113 | 0.000104 | postive |
| GLS | AC239802.2 | 0.453919 | 1.63E-05 | postive |
| GCSH | AC010536.1 | 0.484513 | 3.46E-06 | postive |
| GCSH | AP003068.1 | 0.508352 | 9.28E-07 | postive |
| LIAS | AC008771.1 | 0.430517 | 4.86E-05 | postive |
| PDHB | BX255923.1 | 0.506702 | 1.02E-06 | postive |
| NLRP3 | LINC01678 | 0.51538 | 6.18E-07 | postive |
| DLD | HSD11B1-AS1 | 0.412004 | 0.000109 | postive |
| DBT | AP000759.1 | 0.432256 | 4.49E-05 | postive |
| GCSH | AC002553.2 | 0.423948 | 6.50E-05 | postive |
| ATP7A | TBC1D22A-AS1 | 0.425223 | 6.15E-05 | postive |
| FDX1 | AP000722.1 | 0.602431 | 1.69E-09 | postive |
| LIAS | AC125494.2 | 0.402366 | 0.000163 | postive |
| LIPT1 | AP001056.1 | 0.620551 | 3.91E-10 | postive |
| LIPT1 | CCDC18-AS1 | 0.559735 | 3.77E-08 | postive |
| MTF1 | AC018809.2 | 0.53848 | 1.51E-07 | postive |
| LIPT1 | LINC01521 | 0.600245 | 2.00E-09 | postive |
| LIPT1 | AC010525.1 | 0.486645 | 3.09E-06 | postive |

3. GO

| ONTOLOGY | Description | BgRatio | pvalue | geneID |
| --- | --- | --- | --- | --- |
| BP | tricarboxylic acid cycle | 34/18862 | 1.60E-10 | DLD/PDHA1/PDHB/DLST/DLAT |
| BP | acetyl-CoA metabolic process | 38/18862 | 2.88E-10 | DLD/PDHA1/PDHB/DLST/DLAT |
| BP | acetyl-CoA biosynthetic process from pyruvate | 15/18862 | 9.96E-10 | DLD/PDHA1/PDHB/DLAT |
| BP | acetyl-CoA biosynthetic process | 22/18862 | 5.32E-09 | DLD/PDHA1/PDHB/DLAT |
| BP | aerobic respiration | 86/18862 | 1.94E-08 | DLD/PDHA1/PDHB/DLST/DLAT |
| BP | sulfur compound biosynthetic process | 193/18862 | 2.58E-08 | DLD/PDHA1/PDHB/NFE2L2/LIAS/DLAT |
| BP | sulfur compound metabolic process | 378/18862 | 5.03E-08 | DLD/PDHA1/PDHB/NFE2L2/LIAS/DLST/DLAT |
| BP | acyl-CoA metabolic process | 105/18862 | 5.31E-08 | DLD/PDHA1/PDHB/DLST/DLAT |
| BP | thioester metabolic process | 105/18862 | 5.31E-08 | DLD/PDHA1/PDHB/DLST/DLAT |
| BP | thioester biosynthetic process | 53/18862 | 2.09E-07 | DLD/PDHA1/PDHB/DLAT |
| BP | acyl-CoA biosynthetic process | 53/18862 | 2.09E-07 | DLD/PDHA1/PDHB/DLAT |
| BP | nucleoside bisphosphate metabolic process | 140/18862 | 2.24E-07 | DLD/PDHA1/PDHB/DLST/DLAT |
| BP | ribonucleoside bisphosphate metabolic process | 140/18862 | 2.24E-07 | DLD/PDHA1/PDHB/DLST/DLAT |
| BP | purine nucleoside bisphosphate metabolic process | 140/18862 | 2.24E-07 | DLD/PDHA1/PDHB/DLST/DLAT |
| BP | cellular copper ion homeostasis | 14/18862 | 3.13E-07 | SLC31A1/ATP7A/ATP7B |
| BP | copper ion transport | 16/18862 | 4.81E-07 | SLC31A1/ATP7A/ATP7B |
| BP | nucleoside bisphosphate biosynthetic process | 67/18862 | 5.41E-07 | DLD/PDHA1/PDHB/DLAT |
| BP | ribonucleoside bisphosphate biosynthetic process | 67/18862 | 5.41E-07 | DLD/PDHA1/PDHB/DLAT |
| BP | purine nucleoside bisphosphate biosynthetic process | 67/18862 | 5.41E-07 | DLD/PDHA1/PDHB/DLAT |
| BP | copper ion homeostasis | 17/18862 | 5.84E-07 | SLC31A1/ATP7A/ATP7B |
| BP | cellular respiration | 187/18862 | 9.43E-07 | DLD/PDHA1/PDHB/DLST/DLAT |
| BP | alpha-amino acid metabolic process | 191/18862 | 1.05E-06 | DLD/GCSH/GLS/ATP7A/DLST |
| BP | alpha-amino acid catabolic process | 88/18862 | 1.62E-06 | DLD/GCSH/GLS/DLST |
| BP | cellular amino acid catabolic process | 106/18862 | 3.42E-06 | DLD/GCSH/GLS/DLST |
| BP | organic acid catabolic process | 258/18862 | 4.58E-06 | DLD/GCSH/GLS/DBT/DLST |
| BP | energy derivation by oxidation of organic compounds | 278/18862 | 6.59E-06 | DLD/PDHA1/PDHB/DLST/DLAT |
| BP | response to copper ion | 42/18862 | 9.70E-06 | ATP7A/NFE2L2/ATP7B |
| BP | pyruvate metabolic process | 150/18862 | 1.36E-05 | DLD/PDHA1/PDHB/DLAT |
| BP | cellular amino acid metabolic process | 331/18862 | 1.53E-05 | DLD/GCSH/GLS/ATP7A/DLST |
| BP | purine ribonucleotide biosynthetic process | 175/18862 | 2.49E-05 | DLD/PDHA1/PDHB/DLAT |
| BP | ribonucleotide biosynthetic process | 188/18862 | 3.30E-05 | DLD/PDHA1/PDHB/DLAT |
| BP | peptidyl-lysine modification | 391/18862 | 3.41E-05 | GCSH/LIPT1/ATP7A/LIAS/LIPT2 |
| BP | ribose phosphate biosynthetic process | 195/18862 | 3.80E-05 | DLD/PDHA1/PDHB/DLAT |
| BP | purine nucleotide biosynthetic process | 197/18862 | 3.96E-05 | DLD/PDHA1/PDHB/DLAT |
| BP | purine ribonucleotide metabolic process | 408/18862 | 4.18E-05 | DLD/PDHA1/PDHB/DLST/DLAT |
| BP | purine-containing compound biosynthetic process | 208/18862 | 4.89E-05 | DLD/PDHA1/PDHB/DLAT |
| BP | ribonucleotide metabolic process | 425/18862 | 5.08E-05 | DLD/PDHA1/PDHB/DLST/DLAT |
| BP | small molecule catabolic process | 431/18862 | 5.43E-05 | DLD/GCSH/GLS/DBT/DLST |
| BP | ribose phosphate metabolic process | 435/18862 | 5.68E-05 | DLD/PDHA1/PDHB/DLST/DLAT |
| BP | purine nucleotide metabolic process | 441/18862 | 6.06E-05 | DLD/PDHA1/PDHB/DLST/DLAT |
| BP | lysine catabolic process | 12/18862 | 6.31E-05 | DLD/DLST |
| BP | purine-containing compound metabolic process | 460/18862 | 7.40E-05 | DLD/PDHA1/PDHB/DLST/DLAT |
| BP | lysine metabolic process | 13/18862 | 7.45E-05 | DLD/DLST |
| BP | carboxylic acid catabolic process | 243/18862 | 8.95E-05 | DLD/GCSH/GLS/DLST |
| BP | 2-oxoglutarate metabolic process | 16/18862 | 0.000114 | DLD/DLST |
| BP | dicarboxylic acid metabolic process | 96/18862 | 0.000117 | DLD/GLS/DLST |
| BP | nucleotide biosynthetic process | 264/18862 | 0.000123 | DLD/PDHA1/PDHB/DLAT |
| BP | nucleoside phosphate biosynthetic process | 267/18862 | 0.000129 | DLD/PDHA1/PDHB/DLAT |
| BP | branched-chain amino acid catabolic process | 20/18862 | 0.000181 | DLD/DBT |
| BP | protein maturation | 293/18862 | 0.000184 | GCSH/LIPT1/LIAS/LIPT2 |
| BP | cellular transition metal ion homeostasis | 118/18862 | 0.000215 | SLC31A1/ATP7A/ATP7B |
| BP | aspartate family amino acid catabolic process | 23/18862 | 0.00024 | DLD/DLST |
| BP | branched-chain amino acid metabolic process | 23/18862 | 0.00024 | DLD/DBT |
| BP | removal of superoxide radicals | 23/18862 | 0.00024 | ATP7A/NFE2L2 |
| BP | transition metal ion transport | 127/18862 | 0.000267 | SLC31A1/ATP7A/ATP7B |
| BP | cellular response to oxygen radical | 25/18862 | 0.000284 | ATP7A/NFE2L2 |
| BP | cellular response to superoxide | 25/18862 | 0.000284 | ATP7A/NFE2L2 |
| BP | protein phosphopantetheinylation | 331/18862 | 0.000293 | LIPT1/LIPT2/DLST/DLAT |
| BP | transition metal ion homeostasis | 139/18862 | 0.000348 | SLC31A1/ATP7A/ATP7B |
| BP | response to superoxide | 28/18862 | 0.000358 | ATP7A/NFE2L2 |
| BP | cellular response to copper ion | 28/18862 | 0.000358 | ATP7A/NFE2L2 |
| BP | response to metal ion | 352/18862 | 0.00037 | MTF1/ATP7A/NFE2L2/ATP7B |
| BP | response to oxygen radical | 29/18862 | 0.000384 | ATP7A/NFE2L2 |
| BP | cell redox homeostasis | 43/18862 | 0.000847 | DLD/NFE2L2 |
| BP | vascular associated smooth muscle cell migration | 43/18862 | 0.000847 | ATP7A/NFE2L2 |
| BP | regulation of vascular associated smooth muscle cell migration | 43/18862 | 0.000847 | ATP7A/NFE2L2 |
| BP | cellular response to metal ion | 189/18862 | 0.000852 | MTF1/ATP7A/NFE2L2 |
| BP | response to oxidative stress | 444/18862 | 0.000887 | MTF1/ATP7A/NFE2L2/LIAS |
| BP | metal ion export | 48/18862 | 0.001055 | ATP7A/ATP7B |
| BP | aspartate family amino acid metabolic process | 50/18862 | 0.001144 | DLD/DLST |
| BP | glucose metabolic process | 210/18862 | 0.001156 | PDHA1/PDHB/DLAT |
| BP | cellular response to inorganic substance | 216/18862 | 0.001253 | MTF1/ATP7A/NFE2L2 |
| BP | response to zinc ion | 57/18862 | 0.001484 | MTF1/ATP7A |
| BP | response to cadmium ion | 61/18862 | 0.001698 | MTF1/ATP7A |
| BP | T-helper cell differentiation | 64/18862 | 0.001867 | NLRP3/ATP7A |
| BP | hexose metabolic process | 250/18862 | 0.001905 | PDHA1/PDHB/DLAT |
| BP | CD4-positive, alpha-beta T cell differentiation involved in immune response | 66/18862 | 0.001984 | NLRP3/ATP7A |
| BP | alpha-beta T cell activation involved in immune response | 67/18862 | 0.002044 | NLRP3/ATP7A |
| BP | alpha-beta T cell differentiation involved in immune response | 67/18862 | 0.002044 | NLRP3/ATP7A |
| BP | superoxide metabolic process | 70/18862 | 0.002229 | ATP7A/NFE2L2 |
| BP | positive regulation of response to wounding | 72/18862 | 0.002356 | ATP7A/NFE2L2 |
| BP | T cell differentiation involved in immune response | 73/18862 | 0.002421 | NLRP3/ATP7A |
| BP | monosaccharide metabolic process | 272/18862 | 0.002422 | PDHA1/PDHB/DLAT |
| BP | regulation of muscle cell apoptotic process | 80/18862 | 0.002899 | CDKN2A/NFE2L2 |
| BP | regulation of smooth muscle cell migration | 81/18862 | 0.00297 | ATP7A/NFE2L2 |
| BP | CD4-positive, alpha-beta T cell differentiation | 81/18862 | 0.00297 | NLRP3/ATP7A |
| BP | muscle cell apoptotic process | 84/18862 | 0.00319 | CDKN2A/NFE2L2 |
| BP | aging | 304/18862 | 0.003317 | DLD/CDKN2A/NFE2L2 |
| BP | smooth muscle cell migration | 88/18862 | 0.003495 | ATP7A/NFE2L2 |
| BP | negative regulation of NF-kappaB transcription factor activity | 92/18862 | 0.003813 | NLRP3/CDKN2A |
| BP | CD4-positive, alpha-beta T cell activation | 100/18862 | 0.004487 | NLRP3/ATP7A |
| BP | muscle cell migration | 101/18862 | 0.004575 | ATP7A/NFE2L2 |
| BP | cellular oxidant detoxification | 102/18862 | 0.004663 | ATP7A/NFE2L2 |
| BP | alpha-beta T cell differentiation | 107/18862 | 0.005119 | NLRP3/ATP7A |
| BP | T cell activation involved in immune response | 111/18862 | 0.005497 | NLRP3/ATP7A |
| BP | cellular detoxification | 115/18862 | 0.005889 | ATP7A/NFE2L2 |
| BP | cellular response to toxic substance | 122/18862 | 0.006603 | ATP7A/NFE2L2 |
| BP | detoxification | 138/18862 | 0.008376 | ATP7A/NFE2L2 |
| BP | oxidative phosphorylation | 148/18862 | 0.009581 | DLD/ATP7A |
| BP | alpha-beta T cell activation | 149/18862 | 0.009706 | NLRP3/ATP7A |
| BP | cerebellar Purkinje cell differentiation | 10/18862 | 0.01003 | ATP7A |
| BP | pyramidal neuron differentiation | 10/18862 | 0.01003 | ATP7A |
| BP | serotonin metabolic process | 10/18862 | 0.01003 | ATP7A |
| BP | positive regulation of cell size | 10/18862 | 0.01003 | ATP7A |
| BP | elastic fiber assembly | 10/18862 | 0.01003 | ATP7A |
| BP | response to forskolin | 10/18862 | 0.01003 | FDX1 |
| BP | cellular response to forskolin | 10/18862 | 0.01003 | FDX1 |
| BP | regulation of removal of superoxide radicals | 10/18862 | 0.01003 | NFE2L2 |
| BP | cellular response to reactive oxygen species | 159/18862 | 0.010991 | ATP7A/NFE2L2 |
| BP | suckling behavior | 11/18862 | 0.011028 | GLS |
| BP | regulation of transcription from RNA polymerase II promoter in response to oxidative stress | 11/18862 | 0.011028 | NFE2L2 |
| BP | regulation of T-helper 2 cell differentiation | 11/18862 | 0.011028 | NLRP3 |
| BP | macrophage apoptotic process | 11/18862 | 0.011028 | CDKN2A |
| BP | negative regulation of hematopoietic progenitor cell differentiation | 11/18862 | 0.011028 | NFE2L2 |
| BP | regulation of T-helper 2 cell cytokine production | 11/18862 | 0.011028 | NLRP3 |
| BP | regulation of response to wounding | 164/18862 | 0.01166 | ATP7A/NFE2L2 |
| BP | regulation of respiratory gaseous exchange by nervous system process | 12/18862 | 0.012025 | GLS |
| BP | tryptophan metabolic process | 12/18862 | 0.012025 | ATP7A |
| BP | cerebellar Purkinje cell layer formation | 12/18862 | 0.012025 | ATP7A |
| BP | negative regulation of acute inflammatory response | 13/18862 | 0.01302 | NLRP3 |
| BP | serine family amino acid catabolic process | 13/18862 | 0.01302 | GCSH |
| BP | response to laminar fluid shear stress | 13/18862 | 0.01302 | NFE2L2 |
| BP | T-helper 2 cell cytokine production | 13/18862 | 0.01302 | NLRP3 |
| BP | norepinephrine metabolic process | 13/18862 | 0.01302 | ATP7A |
| BP | regulation of sulfur metabolic process | 13/18862 | 0.01302 | NFE2L2 |
| BP | cellular response to antibiotic | 13/18862 | 0.01302 | ATP7A |
| BP | positive regulation of ERAD pathway | 13/18862 | 0.01302 | NFE2L2 |
| BP | negative regulation of DNA-binding transcription factor activity | 177/18862 | 0.013483 | NLRP3/CDKN2A |
| BP | electron transport chain | 178/18862 | 0.013628 | DLD/FDX1 |
| BP | dicarboxylic acid biosynthetic process | 14/18862 | 0.014015 | GLS |
| BP | regulation of respiratory system process | 14/18862 | 0.014015 | GLS |
| BP | primary amino compound metabolic process | 14/18862 | 0.014015 | ATP7A |
| BP | toxin metabolic process | 15/18862 | 0.015009 | NFE2L2 |
| BP | detoxification of copper ion | 15/18862 | 0.015009 | ATP7A |
| BP | T-helper 2 cell differentiation | 15/18862 | 0.015009 | NLRP3 |
| BP | stress response to copper ion | 15/18862 | 0.015009 | ATP7A |
| BP | lymphocyte activation involved in immune response | 189/18862 | 0.015269 | NLRP3/ATP7A |
| BP | positive regulation of type 2 immune response | 16/18862 | 0.016002 | NLRP3 |
| BP | cerebellar Purkinje cell layer morphogenesis | 16/18862 | 0.016002 | ATP7A |
| BP | indolalkylamine metabolic process | 17/18862 | 0.016994 | ATP7A |
| BP | glutathione biosynthetic process | 17/18862 | 0.016994 | NFE2L2 |
| BP | glutamine family amino acid biosynthetic process | 17/18862 | 0.016994 | GLS |
| BP | positive regulation of smooth muscle cell apoptotic process | 17/18862 | 0.016994 | CDKN2A |
| BP | detoxification of inorganic compound | 17/18862 | 0.016994 | ATP7A |
| BP | replicative senescence | 17/18862 | 0.016994 | CDKN2A |
| CC | mitochondrial matrix | 476/19520 | 1.66E-15 | DLD/FDX1/GCSH/LIPT1/PDHA1/GLS/PDHB/LIAS/LIPT2/DBT/DLST/DLAT |
| CC | oxidoreductase complex | 111/19520 | 7.56E-12 | DLD/GCSH/PDHA1/PDHB/DBT/DLST/DLAT |
| CC | dihydrolipoyl dehydrogenase complex | 10/19520 | 9.34E-08 | DLD/DBT/DLST |
| CC | tricarboxylic acid cycle enzyme complex | 14/19520 | 2.83E-07 | DLD/DBT/DLST |
| CC | mitochondrial protein-containing complex | 265/19520 | 0.00011 | PDHA1/PDHB/DBT/DLAT |
| CC | late endosome | 275/19520 | 0.002268 | SLC31A1/ATP7A/ATP7B |
| MF | oxidoreductase activity, acting on the aldehyde or oxo group of donors, NAD or NADP as acceptor | 36/18337 | 4.75E-08 | DLD/PDHA1/PDHB/DLAT |
| MF | oxidoreductase activity, acting on the aldehyde or oxo group of donors | 44/18337 | 1.09E-07 | DLD/PDHA1/PDHB/DLAT |
| MF | transferase activity, transferring acyl groups other than amino-acyl groups | 225/18337 | 2.69E-06 | LIPT1/LIPT2/DBT/DLST/DLAT |
| MF | transferase activity, transferring acyl groups | 254/18337 | 4.86E-06 | LIPT1/LIPT2/DBT/DLST/DLAT |
| MF | transition metal ion transmembrane transporter activity | 37/18337 | 7.17E-06 | SLC31A1/ATP7A/ATP7B |
| MF | S-acyltransferase activity | 32/18337 | 0.000495 | DLST/DLAT |
| MF | ATPase-coupled transmembrane transporter activity | 58/18337 | 0.001624 | ATP7A/ATP7B |
| MF | copper ion binding | 60/18337 | 0.001737 | ATP7A/ATP7B |
| MF | primary active transmembrane transporter activity | 61/18337 | 0.001795 | ATP7A/ATP7B |
| MF | iron-sulfur cluster binding | 67/18337 | 0.00216 | FDX1/LIAS |
| MF | metal cluster binding | 67/18337 | 0.00216 | FDX1/LIAS |
| MF | acetyltransferase activity | 95/18337 | 0.004288 | DBT/DLAT |
| MF | metal ion transmembrane transporter activity | 425/18337 | 0.00909 | SLC31A1/ATP7A/ATP7B |
| MF | sulfurtransferase activity | 10/18337 | 0.010316 | LIAS |
| MF | cyclin-dependent protein serine/threonine kinase inhibitor activity | 12/18337 | 0.012367 | CDKN2A |
| MF | oxidoreductase activity, acting on a sulfur group of donors, NAD(P) as acceptor | 12/18337 | 0.012367 | DLD |
| MF | peptidoglycan binding | 18/18337 | 0.018496 | NLRP3 |
| MF | active ion transmembrane transporter activity | 206/18337 | 0.018942 | ATP7A/ATP7B |
| MF | 2 iron, 2 sulfur cluster binding | 22/18337 | 0.022562 | FDX1 |
| MF | histone acetyltransferase binding | 27/18337 | 0.027622 | MTF1 |
| MF | sulfur compound binding | 260/18337 | 0.029221 | DLD/DBT |
| MF | NF-kappaB binding | 29/18337 | 0.029639 | CDKN2A |
| MF | RNA polymerase II-specific DNA-binding transcription factor binding | 271/18337 | 0.031538 | CDKN2A/NFE2L2 |
| MF | protein serine/threonine kinase inhibitor activity | 32/18337 | 0.032657 | CDKN2A |

4. KEGG

| Description | BgRatio | pvalue | geneID |
| --- | --- | --- | --- |
| Citrate cycle (TCA cycle) | 30/8145 | 2.87E-09 | DLD/PDHA1/PDHB/DLST/DLAT |
| Carbon metabolism | 115/8145 | 7.58E-08 | DLD/GCSH/PDHA1/PDHB/DLST/DLAT |
| Pyruvate metabolism | 47/8145 | 2.19E-06 | DLD/PDHA1/PDHB/DLAT |
| Glycolysis / Gluconeogenesis | 67/8145 | 9.18E-06 | DLD/PDHA1/PDHB/DLAT |
| Platinum drug resistance | 73/8145 | 1.29E-05 | CDKN2A/SLC31A1/ATP7A/ATP7B |
| Biosynthesis of cofactors | 153/8145 | 0.000235 | DLD/LIPT1/LIAS/LIPT2 |
| Mineral absorption | 60/8145 | 0.00024 | SLC31A1/ATP7A/ATP7B |
| Central carbon metabolism in cancer | 70/8145 | 0.000379 | PDHA1/GLS/PDHB |
| Glyoxylate and dicarboxylate metabolism | 30/8145 | 0.001723 | DLD/GCSH |
| Propanoate metabolism | 32/8145 | 0.00196 | DLD/DBT |
| Glycine, serine and threonine metabolism | 40/8145 | 0.003053 | DLD/GCSH |
| Tryptophan metabolism | 42/8145 | 0.003361 | DLD/DLST |
| Valine, leucine and isoleucine degradation | 48/8145 | 0.004372 | DLD/DBT |
| Lysine degradation | 63/8145 | 0.007431 | DLD/DLST |
| Glucagon signaling pathway | 107/8145 | 0.020448 | PDHA1/PDHB |
| HIF-1 signaling pathway | 109/8145 | 0.021172 | PDHA1/PDHB |

5. uniCox

| gene | HR | HR.95L | HR.95H | pvalue |
| --- | --- | --- | --- | --- |
| LINC02551 | 1.33098 | 1.040634 | 1.702336 | 0.022775 |
| AP001001.1 | 57.98713 | 1.647364 | 2041.144 | 0.025437 |
| AL121749.1 | 0.037704 | 0.002179 | 0.65252 | 0.024231 |
| LINC00665 | 1.347828 | 1.102662 | 1.647505 | 0.003568 |
| AC025741.1 | 3.470379 | 1.80963 | 6.655243 | 0.00018 |
| AC124798.1 | 1.373611 | 1.104432 | 1.708396 | 0.004337 |
| ZNF213-AS1 | 0.147291 | 0.026775 | 0.810255 | 0.027676 |
| AC006033.2 | 0.138943 | 0.029672 | 0.650623 | 0.012223 |
| SNHG8 | 1.25686 | 1.003524 | 1.574149 | 0.046526 |
| AC005277.2 | 1.931563 | 1.363144 | 2.737008 | 0.000214 |
| AL450344.2 | 2.071499 | 1.191713 | 3.60079 | 0.009831 |
| AC092718.3 | 0.010785 | 0.000166 | 0.698781 | 0.033306 |
| AC002116.2 | 1.951044 | 1.219736 | 3.120818 | 0.005291 |
| AL512625.2 | 4.22E-05 | 1.09E-08 | 0.163616 | 0.016875 |
| LINC01060 | 1.51107 | 1.163432 | 1.962585 | 0.00197 |
| AC009495.3 | 1.944149 | 1.209739 | 3.124405 | 0.006022 |
| AP000851.2 | 1.084155 | 1.044375 | 1.125451 | 2.27E-05 |
| LINC00837 | 1.333273 | 1.172271 | 1.516387 | 1.18E-05 |
| DUBR | 0.214984 | 0.054346 | 0.850441 | 0.028462 |
| AL390728.4 | 1.346381 | 1.102497 | 1.644213 | 0.003535 |
| AL133371.2 | 0.689799 | 0.478393 | 0.994627 | 0.046721 |
| SNHG6 | 1.015818 | 1.001584 | 1.030255 | 0.029277 |
| LINC01923 | 1.394748 | 1.035227 | 1.879124 | 0.028699 |
| NSMCE1-DT | 0.010926 | 0.000142 | 0.839951 | 0.041481 |
| AC020911.2 | 1.470491 | 1.054368 | 2.050845 | 0.023093 |
| ERVK-28 | 1.491518 | 1.006696 | 2.209831 | 0.046236 |
| AC004943.2 | 0.40969 | 0.196225 | 0.855373 | 0.017507 |
| AC027801.1 | 2.00455 | 1.180149 | 3.40484 | 0.010089 |
| FAM225B | 0.359111 | 0.161336 | 0.799333 | 0.012121 |
| AC084116.3 | 1.186021 | 1.067036 | 1.318273 | 0.001562 |
| AL031118.1 | 0.041884 | 0.002412 | 0.727324 | 0.029363 |
| IL10RB-DT | 0.108183 | 0.023603 | 0.495849 | 0.004196 |
| AL731567.1 | 5.628376 | 1.495412 | 21.18388 | 0.010618 |
| AC064836.3 | 2.244448 | 1.361965 | 3.698735 | 0.001514 |
| Z99758.1 | 1.239506 | 1.006601 | 1.526299 | 0.043185 |
| RPARP-AS1 | 1.557027 | 1.132815 | 2.140095 | 0.006364 |
| AC090152.1 | 0.470247 | 0.260645 | 0.848404 | 0.012211 |
| AC100821.2 | 3.45826 | 1.744024 | 6.857451 | 0.000382 |
| AC069307.1 | 0.470227 | 0.22529 | 0.981464 | 0.044452 |
| NKILA | 0.685417 | 0.477983 | 0.982873 | 0.039985 |
| AL139241.1 | 7.239033 | 2.039851 | 25.68991 | 0.002191 |
| AL365295.1 | 0.027284 | 0.000755 | 0.986571 | 0.049143 |
| LINC01433 | 2.269638 | 1.110901 | 4.637009 | 0.024545 |
| AC090559.1 | 0.622148 | 0.447039 | 0.865848 | 0.004891 |
| LINC01423 | 0.52179 | 0.281874 | 0.96591 | 0.038419 |
| LINC01678 | 0.139371 | 0.020338 | 0.955086 | 0.044775 |
| AP000722.1 | 1.711342 | 1.195185 | 2.450409 | 0.003352 |

6. model geneCoef

| Gene | Coef |
| --- | --- |
| AC124798.1 | 0.065763 |
| AC006033.2 | -0.10984 |
| AL450344.2 | -0.11527 |
| AL512625.2 | -0.15127 |
| LINC01060 | 0.021444 |
| LINC00837 | 0.179123 |
| AC004943.2 | -0.08469 |
| AC064836.3 | 0.212243 |
| AC100821.2 | 0.054191 |

7. GSEA result

| ID | setSize | enrichmentScore | NES | pvalue |
| --- | --- | --- | --- | --- |
| KEGG_RIBOSOME | 85 | 0.546495 | 1.883775 | 3.27E-05 |
| KEGG_CYTOKINE_CYTOKINE_RECEPTOR_INTERACTION | 175 | -0.50322 | -1.7465 | 3.38E-05 |
| KEGG_CALCIUM_SIGNALING_PATHWAY | 123 | -0.50603 | -1.67903 | 0.000486 |
| KEGG_NEUROACTIVE_LIGAND_RECEPTOR_INTERACTION | 117 | -0.4927 | -1.63133 | 0.002243 |
| KEGG_COMPLEMENT_AND_COAGULATION_CASCADES | 47 | -0.60092 | -1.7334 | 0.002921 |
| KEGG_HEMATOPOIETIC_CELL_LINEAGE | 63 | -0.54789 | -1.67679 | 0.003643 |
| KEGG_JAK_STAT_SIGNALING_PATHWAY | 102 | -0.50016 | -1.63169 | 0.004031 |
| KEGG_PHENYLALANINE_METABOLISM | 13 | 0.73825 | 1.761359 | 0.005089 |
| KEGG_OXIDATIVE_PHOSPHORYLATION | 120 | 0.395913 | 1.456972 | 0.005501 |
| KEGG_DILATED_CARDIOMYOPATHY | 70 | -0.52202 | -1.63023 | 0.008418 |
| KEGG_HYPERTROPHIC_CARDIOMYOPATHY_HCM | 63 | -0.51313 | -1.57041 | 0.012504 |
| KEGG_VIRAL_MYOCARDITIS | 60 | -0.51175 | -1.55577 | 0.020632 |
| KEGG_SYSTEMIC_LUPUS_ERYTHEMATOSUS | 45 | -0.53019 | -1.51729 | 0.029411 |
| KEGG_HEDGEHOG_SIGNALING_PATHWAY | 44 | 0.441714 | 1.405368 | 0.046932 |

8. RT-qPCR

链接：https://pan.baidu.com/s/1jO2eV2BsWBk2tciMYSdrUA?pwd=2222

提取码：2222

--来自百度网盘超级会员V5的分享
